# Molecular Classification of Colorectal Cancer by microRNA Profiling: Correlation with the Consensus Molecular Subtypes (CMS) and Validation of miR-30b Targets

**DOI:** 10.3390/cancers14215175

**Published:** 2022-10-22

**Authors:** Mateo Paz-Cabezas, Tania Calvo-López, Alejandro Romera-Lopez, Daniel Tabas-Madrid, Jesus Ogando, María-Jesús Fernández-Aceñero, Javier Sastre, Alberto Pascual-Montano, Santos Mañes, Eduardo Díaz-Rubio, Beatriz Perez-Villamil

**Affiliations:** 1 Genomics and Microarrays Laboratory, Medical Oncology Department, Instituto de Investigación Sanitaria San Carlos (IdiSSC), Hospital Clinico San Carlos, 28040 Madrid, Spain; 2 Immunology and Oncology Department, Centro Nacional de Biotecnología (CSIC), 28049 Madrid, Spain; 3 Surgical Pathology, Instituto de Investigación Sanitaria San Carlos (IdiSSC), Hospital Clinico San Carlos, 28040 Madrid, Spain

**Keywords:** colorectal cancer, microRNAs, microarray gene-expression profiling, molecular classification, prognostic factors

## Abstract

**Simple Summary:**

Colorectal cancer is one of the most significant causes of cancer mortality worldwide. Patients stratification is central to improve clinical practice and the Consensus Molecular Subtypes (CMS) have been validated as a useful tool to predict both prognosis and treatment response. This is the first study describing that microRNA profiling can define colorectal cancer CMS subtypes as well as mRNA profiling. MicroRNAs small size facilitates its analysis in serum facilitating a real-time analysis of the disease course. Three microRNA subtypes are identified: miR-LS is associated with the low-stroma/CMS2-subtype; miR-MI with the mucinous-MSI/CMS1-subtype and miR-HS with the high-stroma/CMS4-subtype. MicroRNA novel subtypes and association to the CMS classification were externally validated using TGCA data. Analyzing both mRNAs and miRs in the same population enabled identification of miR target genes and altered biological pathways. A miR-mRNA interaction screening and regulatory network selected major miR targets and was functionally validated for the miR30b/SCL6A4 pair.

**Abstract:**

Colorectal cancer consensus molecular subtypes (CMSs) are widely accepted and constitutes the basis for patient stratification to improve clinical practice. We aimed to find whether miRNAs could reproduce molecular subtypes, and to identify miRNA targets associated to the High-stroma/CMS4 subtype. The expression of 939 miRNAs was analyzed in tumors classified in CMS. TALASSO was used to find gene-miRNA interactions. A miR-mRNA regulatory network was constructed using Cytoscape. Candidate gene-miR interactions were validated in 293T cells. Hierarchical-Clustering identified three miRNA tumor subtypes (miR-LS; miR-MI; and miR-HS) which were significantly associated (*p* < 0.001) to the reported mRNA subtypes. miR-LS correlated with the low-stroma/CMS2; miR-MI with the mucinous-MSI/CMS1 and miR-HS with high-stroma/CMS4. MicroRNA tumor subtypes and association to CMSs were validated with TCGA datasets. TALASSO identified 1462 interactions (*p* < 0.05) out of 21,615 found between 176 miRs and 788 genes. Based on the regulatory network, 88 miR-mRNA interactions were selected as candidates. This network was functionally validated for the pair miR-30b/SLC6A6. We found that miR-30b overexpression silenced 3′-UTR-SLC6A6-driven luciferase expression in 293T-cells; mutation of the target sequence in the 3′-UTR-SLC6A6 prevented the miR-30b inhibitory effect. In conclusion CRC subtype classification using a miR-signature might facilitate a real-time analysis of the disease course and treatment response.

## 1. Introduction

Colorectal cancer (CRC) represents a major health problem being the third most frequent cancer and the second cause of cancer death worldwide [1]. CRC is traditionally classified according to clinical and morphological characteristics in TNM stages (American Joint Committee on Cancer). However, the phenotypic diversity of this disease and its clinical behavior are insufficiently explained by the simple histological grade classification and clinical factors in current use. Our group identified four tumor subtypes by transcriptional profiling [2] that largely overlaps in both, subtype distribution and clinic-biological interpretation with the four Consensus Molecular Subtypes (CMS) [3]. Recently relevant reports have confirmed the prognostic and predictive value of CMS subtypes in phase III clinical trials [4,5,6] supporting the use of the CMS classification as a useful tool for patient management. MicroRNAs (miRs) are noncoding small RNAs that regulate gene activity post-transcriptionally. In cancer, they can function as oncogenes or as tumor suppressors, and miR signatures can serve as promising biomarkers [7,8]. Previous attempts to associate miRs and CRC subtypes have identified members of the miR-200 family downregulated in the mesenchymal/CMS4 subtype [9,10]. However, no other associations between specific miRs and the other three tumor subtypes have been described. In this context, using unsupervised hierarchical clustering analysis, we have analyzed miR expression patterns in the CRC samples used in our previous molecular subtyping study [2] to investigate if miRs allowed CRC tumors classification as well as mRNAs. Since one miR can regulate multiple mRNAs, analyzing both mRNAs and miRs in the same population is an excellent strategy to determine miR target genes and identify altered biological pathways and regulatory networks. In this study we report the identification of three miR molecular subtypes that associate to the described CMSs. This can be an important advance, since it would allow the search of the relevant miRs in serum/plasma of patients and their classification, as other authors have reported for pancreatic adenocarcinoma [11], without the need to obtain biopsies or fragments of the tumor, facilitating real-time analysis of the course of the disease and of the response to the treatment. A just released report, develops a miR classifier using supervised analysis to predict four miR subtypes assigned from the four mRNA CMS subtypes [12]. Using in silico machine learning the study of Adam et al. [12] converts the four mRNA-CMS subtypes to four miR-subtypes. This procedure is different than ours. We used unsupervised analysis that does not constrain any subtype number or class. 

## 2. Materials and Methods

### 2.1. Patients and RNA

For this study we have analyzed the same CRC patients’ cohort used for our previous study, including RNA samples [2]. Tumor samples were taken from the Biobank of the Hospital Clinico San Carlos. The study was conducted according to the guidelines of the Declaration of Helsinki, and approved by the Institutional Review Board and Ethics Committee of Hospital Clinico San Carlos. RNA was extracted from fresh frozen tumor samples using TRIZOL and the homogenizer Ultraturrax T8-S8N-5G. RNA quality was measured with Agilent Bioanalyzer 2100. Only tumors with an RNA Integrity Number (RIN) ≥ 6.5 were included in the analysis.

### 2.2. MicroRNA Expression Analysis and Tumor Classification

Agilent miR 21827 microarrays were used to analyze the expression of 939 miRs in 97 CRC tumor samples and 19 normal colon samples. Fluorescence was measured and quantile-normalized using Agilent scanner, Feature Extraction and GeneSpring software. 176 miRs were present in 90% of the samples and therefore considered for the following data analysis. Expression data was median centered and Average-linkage-hierarchical clustering (centered Pearson correlation) was carried out to perform unsupervised tumor classification considering the 176 expressed miRs in the 88 tumor samples from our previous study [2] (complete data set was submitted to ArrayExpress (E-MTAB-9288)). Then, Differential expression between miR subtypes was analyzed using one-way ANOVA, Student Newman-Keuls (SNK) post hoc test and Benjamini-Hochberg multiple test correction. miRs were considered as differentially expressed only if global *p* < 0.05 and fold change > 1.5 considering any of the pairwise subtype comparison.

### 2.3. Identification of miRs Targets and Correlation with mRNA Expression

TALASSO software [13] was used to find miR-mRNA interactions between the 1722 genes selected from our previous study [2] and the 176 expressed miRs. In order to predict miR-target interactions, TALASSO analyzes miRs expression changes and down-regulation of their putative targets. As criteria to select the most relevant miR-transcript interactions, a class comparison analysis was carried out to find differentially expressed genes between groups.

Unpaired Student-*t*-test with Benjamini-Hochberg multiple correction was carried out between normal colon tissue and tumors from the low-stroma, high-stroma and Mucinous-MSI subtypes. Selected genes were considered as differentially expressed at *p* < 0.05 and >1.5-fold expression. Then, miRNA-mRNA predicted interactions were used to construct a regulatory network using Cytoscape software v3.6.1 [14]. Only the largest connected component was considered for each network. Centrality measures were determined using NetworkAnalyzer and CentiScaPe 2.2. Clusters with higher interconnections were unveiled using ClusterViz and EAGLE algorithm with default options (CliqueSize Threshold = 3, ComplexSize Threshold = 2). Two global centrality measurements, radiality and closeness centrality, were considered to rank the most relevant nodes, as they reflect not only the immediate connections of a node (the degree of each node) but the overall structure of the network. Combining two centrality measurers increase the reliability of this kind of approaches to predict the most relevant genes in an interaction network [15]. In our data, those two topological parameters predicted the same upmost central genes, considering that miRNA-mRNA interactions between the 20 upmost central nodes for each subtype were selected as putative candidates, along with the interactions between mRNA and miRNAs involved in the most relevant cluster for each subtype.

Potential microRNA-mRNA interaction candidates were annotated and scored using information from [16] with two different combined validated predicting scores (Weighted Scoring by Precision (WSP) and logistic regression score (LRS)). Previously experimentally validated interactions were determined using four different databases Tarbase (http://www.microrna.gr/tarbase), miRTarBase (http://miRTarBase.mbc.nctu.edu.tw/), miRWalk (http://mirwalk.umm.uni-heidelberg.de) and miRecords (http://miRecords.umn.edu/miRecords), and also with significant Pearson correlation *p*-values from Starbase (http://starbase.sysu.edu.cn/). MicroRNA binding sites were predicted by five different algorithms Pita (https://tools4mirs.org/software/target_prediction/pita/), FindTar (http://bio.sz.tsinghua.edu.cn/findtar/), Miranda (https://www.mirbase.org), rnaHybrid (http://bibiserv.techfak.uni-bielefeld.de/rnahybrid/) and TargetScan (http://genes.mit.edu/targetscan). MicroRNA candidate prioritization was assessed using an automated script, considering that the last accession date, for all databases accession dates, are 21 April 2016. Candidate gene-miR interactions were scored and biologically validated in HEK-293T cell line. 

### 2.4. External Dataset Validation

TCGA data for miRNA and mRNA expression in CRC were downloaded from the repository using TCGA Biolinks [17] package, RNAseq using Illumina HiSeq platform was selected to obtain 285 samples with 20,531 features each. Normalized gene expression data for mRNA was classified in CMS subtypes using CMSclassifier R package 3 according to the nearest CMS criteria. Sample clustering: TCGA raw data for miRNA consisted of 444 samples and 1046 features. Expression data from RNAseq was processed using DESeq2 [18] to obtain normalized counts matrix. Prior to the unsupervised clustering of samples according to miRNA expression, we performed a 3D-PCA visualization to filter out those samples with an outlier expression pattern, following this criterion 5 samples from the initial dataset were excluded. Afterwards, gene features with less than 10 counts in more than 90% of samples were filtered out, resulting in 336 features per sample. Hierarchical clustering on samples was performed using hclust function over log2 transformed normalized expression matrix. Pearson correlation as distance measure and ward linkage as agglomeration method were chosen. In order to create the heatmap visualization, gene features were also classified using the same parameters. Finally, subtype association between miRNA and mRNA classification was addressed using Chi-square test (χ^2^).

### 2.5. MicroRNAs Differentially Expressed between Tumor-Epithelia and Tumor-Stroma

MicroRNAs expression data were downloaded from GSE35602 [19]. Differential expression between the epithelial and stromal components of the tumor was analyzed by T-Test and Benjamini-Hochberg Multiple Correction Test using GeneSpring Dx 14.9 software. Selected miRs were considered as differentially expressed at *p* < 0.05 and >1.5-fold expression between tumor epithelia and tumor stroma.

### 2.6. Evaluation of miRs-Subtypes Using miRaCL20 Classifier

MicroRNAs expression data (miRNA-Seq) from TCGA-COAD were classified using miRaCl classifier [12] available at Github/rsmadam/CMS-miRaCl. Subtype association between miRNA and mRNA classification was determined using Chi-square test (χ^2^).

### 2.7. Cell Lines, Transformation, Transfection and Luciferase Assay

Human HEK-293T cells were grown in Dulbecco’s Modified Eagle Medium (DMEM) supplemented with 10% fetal bovine serum (FBS), penicillin-streptomycin, L-Glutamine and NaPyr in a humidified incubator at 37 °C with 5% of CO_2_. HmiR0133-MR03 (hsa-miR-30b), HmiT070741-MT06 (FAP), HmiT017418a-MT06 (SLC6A6-A), HmiT017418b-MT06 (SLC6A6-B) and miR-Control plasmids from GeneCopoeia were used. XL1-Blue bacteria were transformed by thermal shock and DNA was extracted using Genomed kit (JETSTAR). HEK-293T cells were cultured in triplicate in 24-well plates (0.05 × 106 cells/well). They were transfected with miR-30b and miR-Control, using Lipofectamine 2000 (Invitrogen). Cells were selected with puromycin and miR-30b levels was checked by RT-PCR using Hs03303066_pri (TaqManTMPri-miR Assays) oligonucleotide and U6 as control. HEK-293T-miR30b-expressing cells were transfected with SLC6A6-A, SLC6A6-B or FAP plasmids. Vectors of these plasmids include Firefly and Renilla luciferase reporter genes. After 12 and 24 h Firefly and Renilla luciferases activity were measured using Dual Luciferase Assay Kit (Promega Madison, WI, USA) in a Tecan Infinite 200 Luminometer. Luciferase intensity measurement was performed by triplicate per condition and analyzed as described [20].

### 2.8. Site-Directed Mutagenesis

Predicted miR-30b interaction site at the SLC6A6 3′-UTR (2225-TGTTTAC-2231 nucleotides) was modified using QuikChange site-directed mutagenesis kit (Agilent Technologies, Palo Alto, CA, U.S.A). The oligonucleotide 5′-cctatgagaatctaatgttattacaaagcaggaaagccgccggcc-3′ (2207 to 2251 nucleotides) was designed using QuikChange Primer Design Tool. G2226T, T2228G and C2231A nucleotides were changed to destabilize the predicted interaction with miR-30b.

### 2.9. Statistical Analysis

Luciferase analysis results were analyzed using Student’s *t*-test to compare mutated vs control mir-30b. Subtype association was addressed using χ^2^ Chi-square test. In order to compare the distribution of qualitative variables between groups Fisher exact test was applied (as all the variables presented less than 5 events in at least one of the categories) and “Mantel-Haenzel Test” for b-catenin linear categories. Mean comparison of quantitative variables between subtypes was performed using Kruskal-Wallis test. Statistical analysis was performed using GraphPad Prism 6 and R software.

## 3. Results

### 3.1. Tumor Classification Based on miR Expression Patterns and Association to mRNA Subtypes

**Figure 1:**
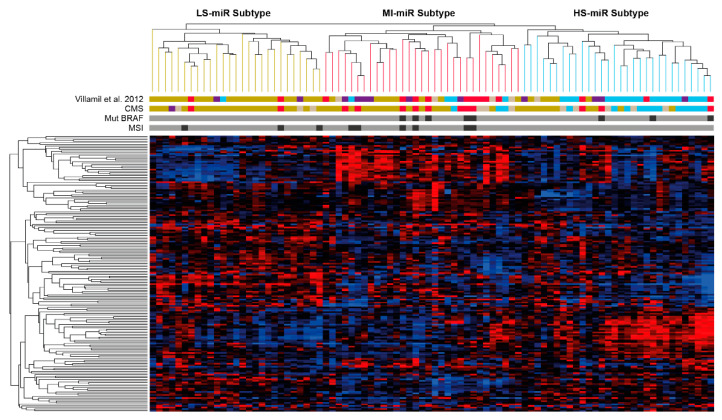
Molecular classification of tumors and miRs. Centered Pearson correlation and average-linkage-hierarchical clustering of the 88 tumor samples and 176 miRs in three miR tumor subtypes (miR-LS pistachio-green lines; miR-MI red lines and miR-HS light blue lines). Villamil et al. subtypes, CMSs, BRAF mutations and MSI are specified below the tree. Low-stroma-subtype/CMS2: pistachio green bar; mucinous-MSI-subtype/CMS1: red bar; high-stroma-subtype/CMS4: light blue; immunoglobulin-related: purple bar; unclassified samples: beige bar. Black bar: BRAF mutated and MSI; grey bar: BRAF wt and MSS. Heatmap intensities: 3.099 (red) to −3.099 (dark blue).

MicroRNAs arranged tumor samples in three clusters (Figure 1). There is a significant association (*p* < 0.001) of the three miR subtypes with the four mRNA subtypes identified by us [2] as well as with the CMS subtypes [3] (Table 1). Supplementary Table S1 shows the classification of the 88 tumors from our previous study [2] using the SSP and RF [3]. miR-Cluster-1 contains 27 tumors showing a higher proportion of tumors belonging to the low-stroma-subtype, as well as the lowest proportion of stromal component in the tumors; consequently, we named this subtype miR-LS (miR-Low-Stroma). Additionally, miR-LS show a significant association with CMS2 whether random forest (RF) or single sample predictor (SSP) were used for sample classification. The highest proportion of tumors from the mucinous-MSI-subtype as well as from CMS1 are in miR-cluster-2 which contains 31 tumors; mucinous histology as well as microsatellite instability (MSI) are associated to this cluster, accordingly we term this cluster miR-MI (Mucinous, Instable). Cluster-3 with 30 tumors contains the highest proportion of tumors of the high-stroma-subtype as well as the highest proportion of stroma in the tumors; we term this cluster miR-HS (High Stroma). Like-wise, the highest proportion of tumors classified as CMS4 associate to miR-HS subtype.

### 3.2. External Dataset Validation

CRC data from TCGA were classified according to the CMS subtypes, resulting in the following subtype distribution for the 285 samples: CMS1 (59), CMS2 (144), CMS3 (33) and CMS4 (49). Hierarchical clustering of miRNA expression (Supplementary Figure S1) unveiled three different groups according to miR expression with the following correspondence with CMS subtypes determined by mRNA expression (Supplementary Table S2), this association presented a significant correlation (*p* < 0.0001) and was performed in those 228 samples with mRNA and miRNA data.

### 3.3. Comparison between miR-LS, miR-HS and miR-MI with the miRCL20 Classifier Subtypes

Association between unsupervised miRNA subtypes (miR-LS, miR-HS, miR-MI) and miRaCl20 (CMS subtyping using miRNA data) was addressed in both TCGA data and Agilent CRC miRNA microarray dataset.

CMS distribution in TCGA data according to miRaCl20 supervised classifier resulted in 41 CMS1, 90 CMS2, 22 CMS3, 73 CMS4 and 2 unclassified for the total 228 samples. In the case of the microarray dataset samples were distributed: 23 CMS1, 44 CMS2, 9 CMS3 and 12 CMS4 for the 88 samples. 

Association between the three miR subtypes (miR-LS, miR-HS, miR-MI) and miRaCl CMS subtypes (Supplementary Figures S2 and S3) is significant resulting pvalue of Chi-square test (χ^2^) was < 2 × 10^−16^ in both cases, with a wider consensus in high-stroma- and low-stroma- subtypes (CMS4 and CMS2). 

### 3.4. Stromal or Epithelial Localization of the miRs Differentially Expressed between Subtypes

Stroma proportion is associated to miR-subtypes (Table 1) but our study was not designed to distinguish miR expression between the stromal or epithelial components of the tumor. To find the contribution of stroma or epithelia to miR expression we took advantage of the study of Nishida et al. [19] in which miR expression was specifically analyzed using laser microdissection, in tumor stroma and in tumor epithelia. From the 176 miRs selected for tumor classification, 45 miRs were significantly differentially expressed at *p* < 0.05 and FC > 1.5 between tumor epithelia and tumor stroma. 

The 176 miRs were also arranged in clusters. Among all, three of them showed the most significant miRs differentially expressed between clusters. Interestingly, two of these clusters contained miRs differentially expressed between tumor-stroma and tumor-epithelia as well (Supplementary Table S3). 

**Figure 2:**
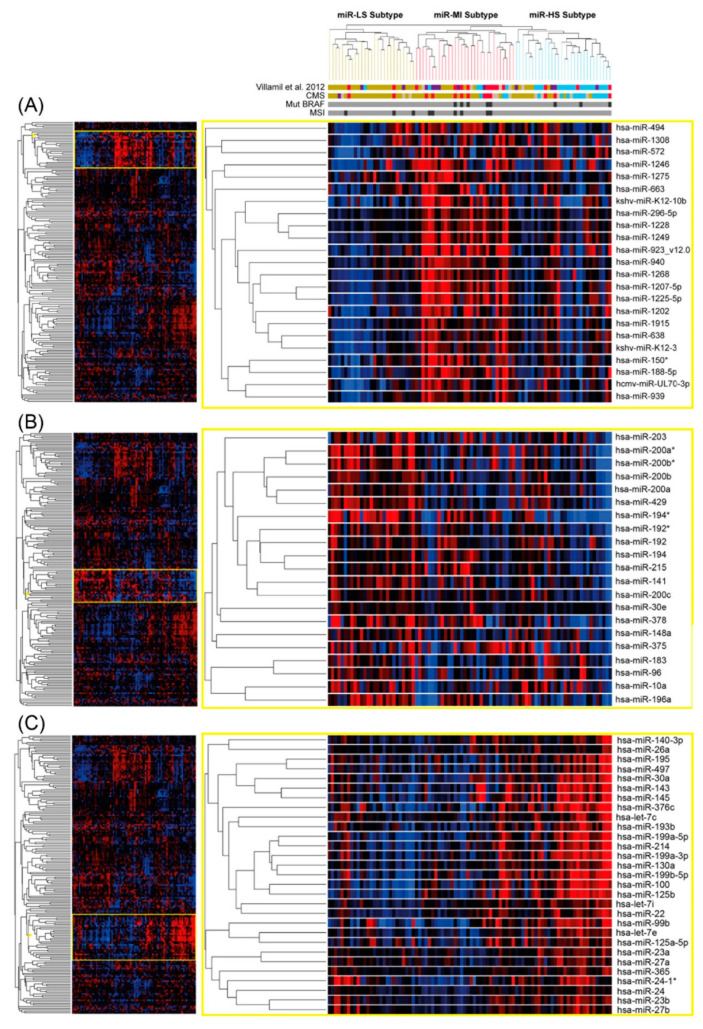
MicroRNAs distribution between Subtypes. (**A**) miRNA-Cluster-A: miRs down-regulated in the miR-LS-subtype and up-regulated in the miR-MI-subtype (23 miRs located in the heatmap between the 7th and the 29th miRs). (**B**) miRNA-Cluster-B: miRs inhibited in the miR-HS-subtype (21 miRs located in the heatmap between the 89th and the 109th miRs). (**C**) miRNA-Cluster-C: miRs upregulated in the miR-HS-subtype (29 miRs located in the heatmap between the 114th and the 142nd miRs). Heatmap intensities: 3.099 (red) to −3.099 (dark blue).

MicroRNA-Cluster-A contains miRs that are down-regulated mainly in the miR-LS-subtype and up-regulated in the miR-MI-subtype (Figure 2A). It is worth noting that among the miRs of this cluster are viral miRs such as the human cytomegalovirus-encoded miR, hcmv-miR-UL70-3p and the Kaposi’s sarcoma-associated herpesvirus miRs: kshv-miR-K12-3 and kshv-miR-K12-10b. Other relevant miRs of this cluster that have been shown to be involved in CRC progression are miR-572 [21], miR-1246 [22], and miR-494 [23]. This group of miRs does not show particularly a specific stromal or epithelial localization (Supplementary Table S3).

MicroRNA-Cluster-B contains miRs that are particularly inhibited in the miR-HS-subtype (Figure 2B). miR-141; miR-200a; miR-200b; miR-200c and miR-429 are in this cluster and belong to the miR-200 family. Other relevant miRs down-regulated in this cluster are miR-378 and miR-194. The miRs of this cluster are down-regulated in the stroma and up-regulated in the epithelia (Supplementary Table S3).

MicroRNA-Cluster-C contains miRs that are upregulated in the miR-HS-subtype (Figure 2C). Members of the miR-30 family and of the miR-100 family such as miR-100, miR-125 and miR-99 are in microRNA-Cluster-C. Other relevant miRs of this cluster are miR-143 and miR-145. These miRs are up-regulated in the stroma and down-regulated in the epithelia (Supplementary Table S3).

### 3.5. Identification of miRs Targets, Selection of Relevant Interactions Associated to Subtypes and Altered Pathways

TALASSO software [13] identified 1462 significant (*p* < 0.05) interactions between 176 miRs and 788 genes out of the 21615 putative interactions (Supplementary Table S4). Out of the 788 genes showing significant miR interactions, 166 genes were differentially expressed in Low-stroma/subtype, 158 in High-stroma/subtype and 78 in Mucinous-MSI/subtype.

In order to identify relevant targets in miR-mRNA interaction patterns, three subtype specific network graphs were generated using those predicted interactions with differential expression (Supplementary Figure S4). MicroRNAs and mRNAs were represented as nodes, connected according to the in-silico predicted interactions (*p* < 0.05), topological parameters and selecting criteria for the obtained networks are available in Supplementary Table S5.

**Figure 3:**
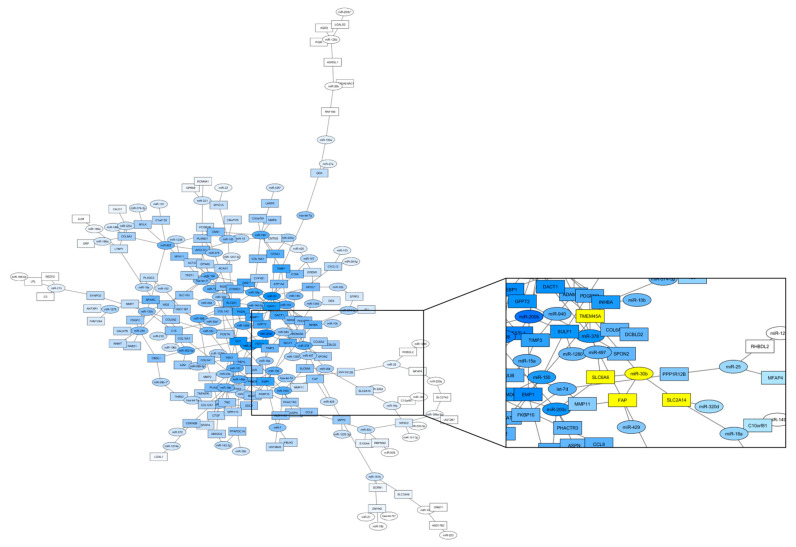
Regulatory Network of the High-Stroma/CMS4-miR-HS-subtype. Nodes reflect mRNAS (squares) and miRNAS (circles), while edges represent a predicted interaction between them. Grey intensity is mapped to each node’s closeness centrality value, the lighter nodes being the most marginal nodes. Square details the interactions between miR-30b and their first neighbors (those genes with a predicted interaction) represented as striped squares.

A list of 88 mRNA-miR interactions was annotated and ranked (Supplementary Table S6), After discarding those interactions that were already biologically validated and taking in consideration the observed expression profiles between subtypes, network centrality values and annotated scores for each interaction, we decided to focus on studying miR-30b-FAP and miR-30b-SLC6A6 interactions as final candidates for biological validation. Moreover, miR-30b and their targets (FAP and SLC6A6) belong to the most connected cluster in miR-HS interaction network (Figure 3), being the subtype featuring the lowest survival.

### 3.6. SCL6A6 Up-Regulated in the High-Stroma/CMS4 Subtype Shows Specific Interaction with miR-30b In Vitro

**Figure 4:**
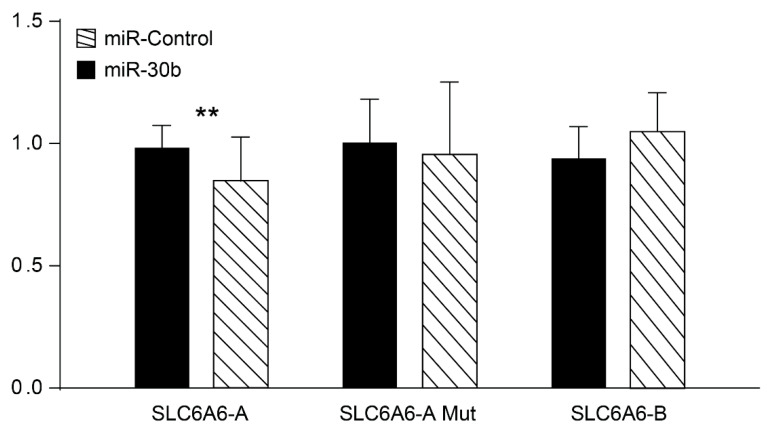
miR-30b target SLC6A6 expression. Luciferase reporter assays of HEK-293 cells transduced with pre-miR-30b or a control miR, and transfected with the 3′-UTR of SLC6A6-A wild-type, SLC6A6-A mutated in the target sequence, or SLC6A6-B lacking the predicted miR-30b binding site. Luciferase activity was normalized to that of control-transfected cells. Data shown as mean ± SEM of triplicates per experiment (*n* = 3). ** *p* < 0.005.

The genes SLC6A6 and FAP, that are up-regulated in the High-stroma/CMS4 subtype, show in-silico interaction with miR-30b (Figure 3) which is down-regulated in tumors and in the stroma versus the epithelia component of the tumor (Supplementary Table S3). In order to validate in-silico predicted miR-transcript interactions, HEK-293T cells were transfected with a miR-30b expression plasmid and with reporter plasmids containing 3′UTR regions of the genes SLC6A6 and FAP. Since SLC6A6 3′UTR region is too long, two different reporter plasmids were used SLC6A6-A (between 2174 and 4573) carrying the putative miR-30b binding site (2225-TGTTTAC-2231) and SLC6A6-B (from 4353 to 6528 nucleotide). MicroRNA-30b significantly (*p* = 0.0038) decrease luciferase activity of the SLC6A6-A reporter plasmid. No significant differences in luciferase activity were found when the putative binding site in SLC6A6-A is mutated or when plasmid SLC6A6-B lacking miR-30b predicted binding site is used (Figure 4). When using FAP 3′UTR reporter plasmid, no differences were found between miR-30b and miR-Control (not shown). These results indicate that miR-30b binds to SLC6A6 3′UTR region to decrease SLC6A6 3′UTR-driven reporter expression.

## 4. Discussion

Tumor molecular classification using unsupervised analysis of gene expression is a powerful tool that has been widely applied to distinguish tumor subgroups with shared biological programs and similar clinical behavior [24]. In contrast, tumor subtyping using unsupervised analysis of miR expression has been barely employed. MicroRNAs are shown to regulate gene expression, and both, miR and mRNA expression patterns are altered in cancer [8]. Since miRs and mRNAs coordinately regulate pathways involved in CRC, our hypothesis was that miR profiling, could also classify CRC in molecular subtypes and that these miR-subtypes, would probably correlate with the described mRNA tumor subtypes [2,3]. In this report we describe the identification of three tumor subtypes based on miR expression patterns that correlates significantly with the four tumor subtypes previously discovered [2,3]. Subtype miR-LS is associated with low-stroma-subtype and CMS2, miR-MI is associated with mucinous-MSI-subtype and CMS1 and miR-HS is associated with high-stroma-subtype and CMS4. Consequently, it is feasible to classify colorectal tumors in the described molecular subtypes by miRs expression profiling. As expected, this correlation is also maintained when the CMS are assigned using miRNA data through miRaCl classifier. Despite of being two strategies for miR classification, they differ in their approach; whereas miRaCl classifier is a supervised method to determine the CMS subtype according to the miR data, our classification does not take any kind of previous assumption to segregate samples. Despite those differences, both classifications have a high correlation in high and low stroma subtypes, supporting the continuous flow of evidence of the role of stroma in the course of the colorectal disease. 

This could be an important advance, since it would allow in future the search of these miRs in plasma and the classification of patients without the need to obtain tumor fragments, facilitating real-time analysis of the course of the disease and the response to treatment, as has been described for pancreatic adenocarcinoma [11].

MicroRNA-Cluster-A contains miRs that are up-regulated in the miR-MI-subtype and shows high expression of miRs belonging to the herpesvirus family [25,26]. It has been shown that viral miRs are able to modulate innate immune responses. MSI and CMS1 tumors are characterized by a higher level of tumor-infiltrating lymphocytes and activation of immune evasion pathways. MicroRNA-Cluster-B contains miRs down-regulated in the miR-HS-subtype. These miRs have been shown to be down-regulated in the stroma compared to the epithelia component of the tumor [19] as well. MicroRNA-Cluster-B is mainly composed with members of the miR-200 family. It has been reported that methylation of the miR-200 promoter identifies tumors of the CMS4 [9,10] this agrees with the lower expression of these miRs in the miR-HS-subtype we found in our results. Other relevant miRs down-regulated in this cluster are miR-378 and miR-194. Low levels of miR-378 and miR-194 are implicated in the malignant phenotype of CRC and restoration of their expression inhibits EMT (Epithelial-Mesenchymal Transition) and prevent the migration and invasion of colon cancer cells [27,28]. 

MicroRNA-Cluster-C contains members of the miR-30 family and of the miR-100 family and are up-regulated in the miR-HS-subtype. Interestingly miRs of this cluster have been reported to be up-regulated in the stroma and down-regulated in the epithelia component of the tumor [19]. Overexpression of miR-100 and miR-125b has been associated with resistance to cetuximab treatment [29]. Other relevant miRs of this cluster are miR-143 and miR-145, these miRs are frequently reduced in colon cancer [30]. We found that when compared with normal colon tissue, tumor miR-143 and miR-145 levels are down regulated around three-fold in clusters miR-LS and miR-MI. However, miR-143 and miR-145 are up-regulated in miR-HS subtype when compared to the other miR clusters but still inhibited with respect to normal colon tissue. An elegant report shows that miR-143 and miR-145 are expressed in the intestinal mesenchyme [31]; this could explain the higher miR-143 and miR-145 in the miR-HS-subtype.

Appropriate integration of miR and mRNA expression profiles is essential to properly understand regulatory pathways and cellular dysfunction in CRC. Elucidating miR targets by bioinformatic analysis permits the identification of a panoply of miR-mRNA possible interactions that need to be ranked. Network analysis is an excellent tool for the selection of the most significant miR-mRNA interactions. Although relevant nodes were found in the three subtypes analyzed (low-stroma, high-stroma and Mucinous-MSI) for in vitro validation we focused on the high-stroma-subtype associated with a poor clinical outcome. The best scores within non-biologically-validated interactions were obtained between miR-30b which is down-regulated in the miR-HS subtype, and FAP or SLC6A6 genes, which are up-regulated in the high-stroma-subtype. High FAP and SLC6A6 levels are associated with worse prognosis in CRC [32,33]. We could not biologically validate miR-30b and FAP interaction; however, miR-30b has been shown to silence SLC6A6 expression. Since higher levels of SLC6A6 are associated with maintenance of stem-cells properties and with chemoresistance [33] restoring miR-30b could be a promising strategy for the treatment of CRC patients of the High-stroma/CMS4 subtype.

## 5. Conclusions

In summary, we show that miR profiles classify colorectal tumors with a straight correlation with the molecular subtypes identified by transcriptional profiling. miR-LS is associated with low-stroma/CMS2, miR-MI with the mucinous-MSI/CMS1 and miR-HS with high-stroma/CMS4 subtypes. Furthermore, the miR/mRNA network identified in High-stroma/CMS4 subtype was validated for the miR30b/SCL6A4 pair. Considering this, using miRs as a classifier provides a promising scenario, the classification of colorectal cancer patients by miR determination in plasma, allowing the classification of patients avoiding invasive procedures and allowing real-time analysis of the course of the disease and response to treatment by liquid biopsy. 

## Figures and Tables

**Table 1:** Association of miRNA clusters to Villamil et al. subtypes, to CMS and to clinic-biological parameters.

		miR-LS (*n* = 27)	miR-MI (*n* = 30)	miR-HS (*n* = 31)	*p* Value
Villamil et al., 2012 Subtypes	Low Stroma	22	7	6	0.000 χ^2^
	Immunoglobul	2	6	4	
	High Stroma	1	4	17	
	Mucinous-MSI	2	9	3	
	Unclassified	0	4	1	
RF	CMS1	1	7	2	0.000 χ^2^
	CMS2	15	9	10	
	CMS3	4	0	0	
	CMS4	0	3	13	
	NA	7	11	6	
SSP	CMS1	2	8	3	0.001 χ^2^
	CMS2	21	16	11	
	CMS3	1	0	0	
	CMS4	0	3	12	
	NA	3	3	5	
Microsatellite	MSS	24	24	31	0.036 χ^2^
	MSI	3	6	0	
Histologic type	Conventional	26	24	28	0.144 χ^2^
	Mucinous	1	6	3	
BRAF	WT	27	25	28	0.091 χ^2^
	Mut	0	5	3	
FF Stroma	Range	(5–28)	(5–40)	(8–65)	0.000 ^KW^
	Median	7.5	13.75	22.5	
FFPE Stroma	Range	(5–20)	(5–40)	(5–60)	0.004 ^KW^
	Median	10	10	20	

RF: Random Forest, SSP: single sample predictor. FF: Fresh-Frozen, FFPE: Formalin Fixed Paraffin embedded. ^KW^: Kruskal Wallis, χ^2^: Chi-Squared test.

## Data Availability

ArrayExpress E-MTAB-9288.

## Supplementary Materials

The following supporting information can be downloaded at: https://www.mdpi.com/article/10.3390/cancers14215175/s1, Table S1: Association of Villamil et al. 2012 Subtypes and CMS; Table S2: miR vs CMS subtypes in TCGA; Table S3: miRs differentially expressed between clusters; Table S4: TALASSO Interaction between miRs and target genes; Table S5: Topological parameters of regulatory networks; Table S6: Final interactions; Figure S1: Hierarchical Clustering of miR expression from the TCGA dataset; Figure S2: Association between miR subtypes (miR-LS; miR-MI; miR-HS) and miRaCL20A (miRNA CMS); Figure S3: TCGA: Association between miR subtypes (miR-LS; miR-MI; miR-HS) and miRaclCL20 (CMS); Figure S4: Regulatory networks of miRNA-mRNA interactions in each CRC tumor subtype.
