# Supplementary material for: Molecular Classification of Colorectal Cancer by microRNA Profiling: Correlation with the Consensus Molecular Subtypes (CMS) and Validation of miR-30b Targets
Source: Cancers (Basel). 2022 Oct 22;14(21):5175. doi: 10.3390/cancers14215175 (PMC9656292; doi:10.3390/cancers14215175)
Supplement: Supplementary file 1 [file cancers-14-05175-s001.zip › cancers-1966190-supplementary/cancers-1966190-supple-figures.pdf]

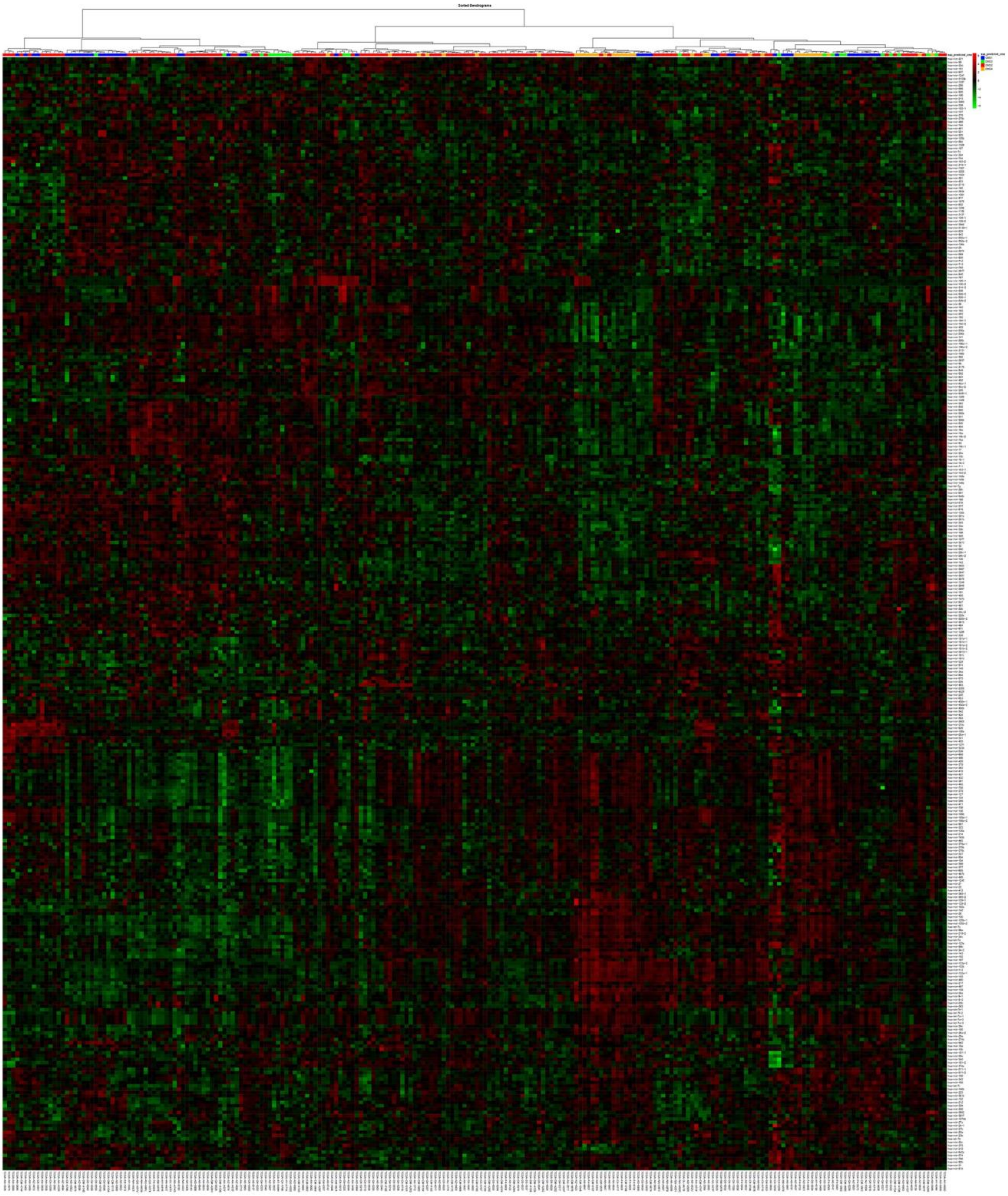

Figure S1. Hierarchical Clustering of miR expression from the TCGA datase.

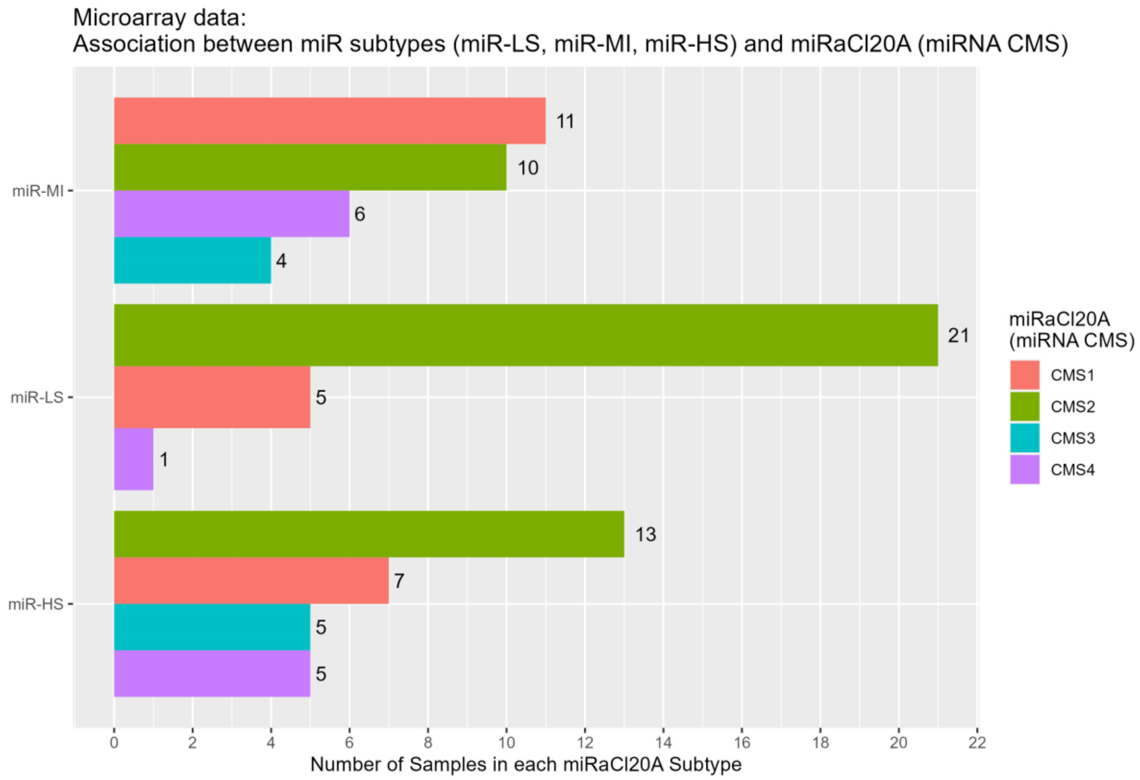

Figure S2. Association between miR subtypes (miR-LS; miR-MI; miR-HS) and miRaCL20A (miRNA CMS).

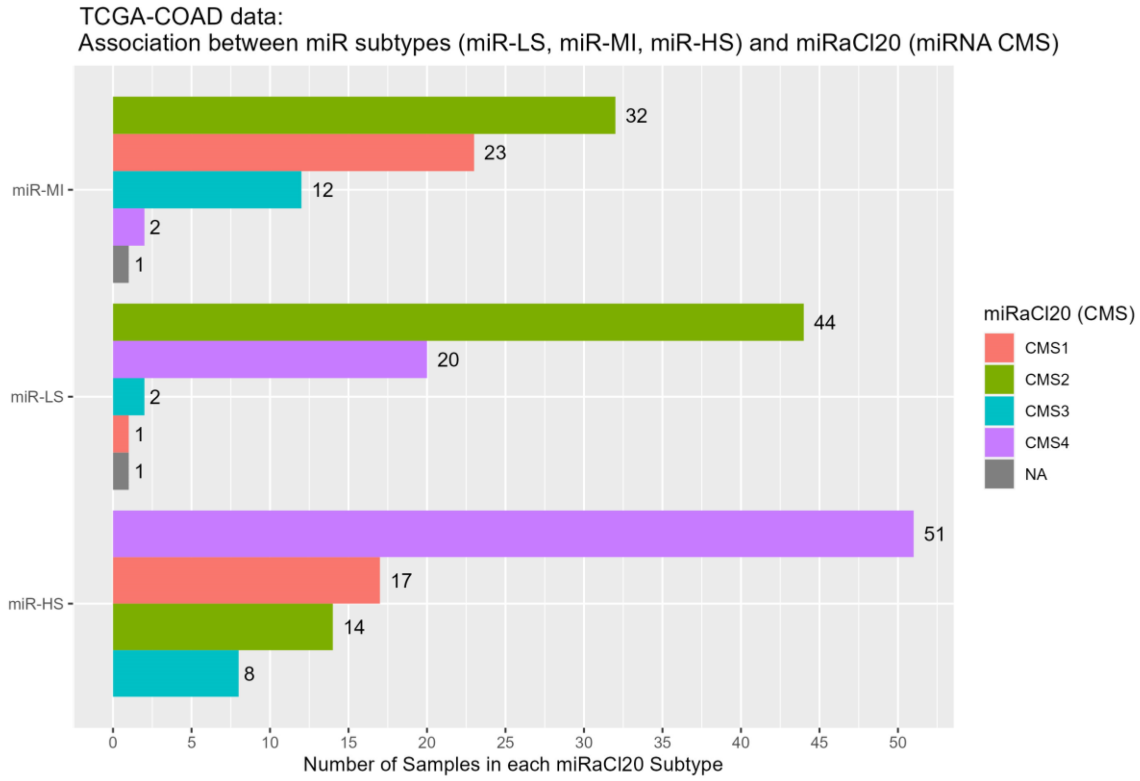

Figure S3. TCGA: Association between miR subtypes (miR-LS; miR-MI; miR-HS) and miRaclCL20 (CMS).

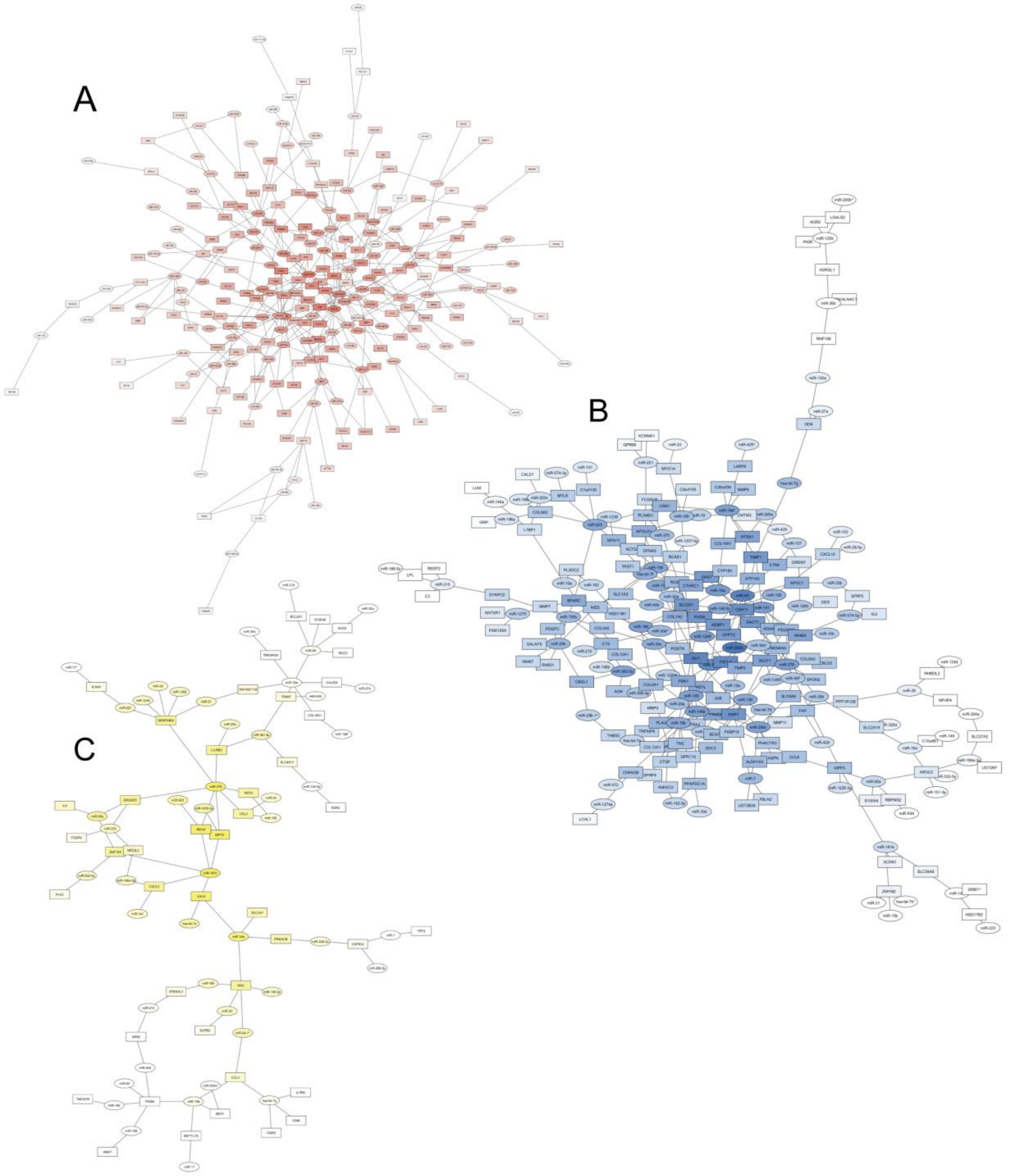

Figure S4. Regulatory networks of miRNA-mRNA interactions in each CRC tumor subtype. (A) low stroma subtype; (B) high stroma subtype; and (C) mucinous and unstable subtype. Nodes reflect mRNAs (squares) and miRNAs (circles), while edges represent a predicted interaction between them. Colour intensity is mapped to each node closeness centrality value, being the lighter nodes the most marginal nodes. Networks A and B are similar in terms of topological parameters, while the network C has less nodes and is less interconnected.
