# Supplementary material for: Molecular Classification of Colorectal Cancer by microRNA Profiling: Correlation with the Consensus Molecular Subtypes (CMS) and Validation of miR-30b Targets
Source: Cancers (Basel). 2022 Oct 22;14(21):5175. doi: 10.3390/cancers14215175 (PMC9656292; doi:10.3390/cancers14215175)
Supplement: Supplementary file 1 [file cancers-14-05175-s001.zip › cancers-1966190-supplementary/Supplementary Table S1.pdf]

Supplementary Table S1. Association of Villamil et al. 2012 Subtypes and CMS

		Low Stroma (n=35)	Immunrel. (n=12)	High Stroma (n=22)	Mucinous-MSI (n=14)	Unclass (n=5)	Total (n=88)	pvalue
SSP	CMS1	0	4	0	9	0	13	$0.000^{\chi 2}$
	CMS2	32	6	5	1	4	48	
	CMS3	1	0	0	0	0	1	
	CMS4	0	1	12	2	0	15	
	NA	2	1	5	2	1	11	
RF	CMS1	0	4	0	6	0	10	$0.000^{\chi 2}$
	CMS2	25	4	3	0	2	34	
	CMS3	4	0	0	0	0	4	
	CMS4	0	1	13	2	0	16	
	NA	6	3	6	6	3	24	

RF: Random Forest, SSP: single sample predictor. χ2: Squared Chi.
