# Supplementary material for: Molecular Classification of Colorectal Cancer by microRNA Profiling: Correlation with the Consensus Molecular Subtypes (CMS) and Validation of miR-30b Targets
Source: Cancers (Basel). 2022 Oct 22;14(21):5175. doi: 10.3390/cancers14215175 (PMC9656292; doi:10.3390/cancers14215175)
Supplement: Supplementary file 1 [file cancers-14-05175-s001.zip › cancers-1966190-supplementary/Supplementary Table S2.pdf]

Supplementary Table S2. miR vs CMS subtypes in TCGA

		miR-MI	miR-LS	miR-HS	Total	pvalue
**SSP**	CMS1	23	2	21	46	$3 \cdot 10^{-12}$ $^{\chi 2}$
	CMS2	37	57	30	124	
	CMS3	10	2	11	23	
	CMS4	0	7	28	35	
	Total	70	68	90	228	

SSP: single sample predictor. $\chi 2$: Squared Chi.
