# Supplementary material for: Molecular Classification of Colorectal Cancer by microRNA Profiling: Correlation with the Consensus Molecular Subtypes (CMS) and Validation of miR-30b Targets
Source: Cancers (Basel). 2022 Oct 22;14(21):5175. doi: 10.3390/cancers14215175 (PMC9656292; doi:10.3390/cancers14215175)
Supplement: Supplementary file 1 [file cancers-14-05175-s001.zip › cancers-1966190-supplementary/Supplementary Table S3.pdf]

Supplementary Table S3. miRs differentially expressed between clusters

microRNA-Cluster-A	Nishida Epithelia/ stroma	p(Corr)global	p[LS]vs[MI]	FC[LS]vs[MI]	p[MI]vs[HS]	FC[MI]vs[HS]	p[LS]vs[HS]	FC[LS]vs[HS]
hsa-miR-494	NA	4,45E-03	3,63E-03	-1,8238838	0,008900761	1,6204097	NS	-1,1255695
hsa-miR-1308	NA	2,88E-02	1,65E-02	-1,6728566	NS	1,1912748	NS	-1,4042575
hsa-miR-572	NA	6,95E-03	6,46E-03	-2,827809	NS	-1,1454176	0,00614185	-3,239022
hsa-miR-1246	NA	1,62E-04	1,63E-04	-2,7361138	0,00125315	2,122668	NS	-1,2889974
hsa-miR-1275	NA	5,35E-05	8,03E-04	-1,4807942	0,000128456	1,6954168	NS	1,1449375
hsa-miR-663	up	2,61E-02	4,20E-02	-1,4368114	0,023249011	1,603037	NS	1,1156906
kshv-miR-K12-10b	NA	1,05E-05	1,09E-04	-3,8913825	0,000259624	2,7672162	NS	-1,4062445
hsa-miR-296-5p	up	1,01E-08	1,07E-04	-1,6572812	0,00011427	1,5793369	NS	-1,0493525
hsa-miR-1228	NA	7,75E-10	1,07E-04	-1,7305673	0,000114256	1,6395206	NS	-1,0555325
hsa-miR-1249	NA	7,75E-10	1,07E-04	-2,2511992	0,00011432	1,7958544	0,02869284	-1,2535533
hsa-miR-923_v12.0	NA	3,79E-06	1,08E-04	-2,634973	0,000161488	2,1092162	NS	-1,2492664
hsa-miR-940	NA	9,55E-09	1,14E-04	-1,7054332	0,000107176	1,7259907	NS	1,0120541
hsa-miR-1268	NA	1,32E-06	1,07E-04	-2,2168941	0,000144883	1,8100151	NS	-1,2247932
hsa-miR-1207-5p	NA	7,75E-10	1,07E-04	-2,6335902	0,000114281	2,0964613	NS	-1,2562073
hsa-miR-1225-5p	up	7,75E-10	1,07E-04	-3,238272	0,000114448	2,2634692	0,01720211	-1,4306675
hsa-miR-1202	NA	3,66E-06	1,07E-04	-2,8089678	0,001144121	1,8237784	0,01666644	-1,5401914
hsa-miR-1915	NA	1,11E-06	1,07E-04	-1,8946868	0,000193451	1,5481112	NS	-1,2238699
hsa-miR-638	NA	9,18E-08	1,07E-04	-2,3603992	0,000480387	1,5816162	0,00188560	-1,4923971
kshv-miR-K12-3	NA	1,05E-06	1,07E-04	-2,2222123	0,001178319	1,5434484	0,00564838	-1,4397709
hsa-miR-150*	NA	1,68E-03	1,14E-03	-2,5658236	0,004923649	2,0846083	NS	-1,2308421
hsa-miR-188-5p	up	7,67E-06	1,08E-04	-3,2322237	0,000819174	2,0982552	0,04330461	-1,5404341
hcmv-miR-UL70-3p	NA	4,47E-04	1,91E-04	-5,526676	NS	2,147697	0,01711591	-2,5733037
hsa-miR-939	NA	2,59E-03	1,51E-03	-2,2586336	0,009768185	1,8133718	NS	-1,2455436

microRNA-Cluster-B	Nishida Epithelia/ Stroma	p(Corr)global	p[LS]vs[MI]	FC[LS]vs[MI]	p[MI]vs[HS]	FC[MI]vs[HS]	p[LS]vs[HS]	FC[LS]vs[HS]
hsa-miR-10a	NA	2,00E-04	1,77E-04	2,1243937	0,00148410	-1,7527252	NS	1,2120516
hsa-miR-141	up	2,23E-03	4,86E-02	1,3576608	NS	1,3337342	0,00068065	1,8107586
hsa-miR-148a	up	NA	NA	NA	NA	NA	NA	NA
hsa-miR-183	up	1,61E-03	8,01E-04	1,7912791	0,00800363	-1,5128536	NS	1,1840398
hsa-miR-192	up	NA	NA	NA	NA	NA	NA	NA
hsa-miR-192*	up	2,27E-03	4,17E-02	1,4960238	NS	1,4199195	0,00071871	2,1242335
hsa-miR-194	up	6,23E-03	NS	1,1415815	0,01888029	1,396252	0,00355834	1,5939357
hsa-miR-194*	up	6,23E-03	NS	1,1415815	0,01888029	1,396252	0,00355834	1,5939357
hsa-miR-196a	up	5,05E-03	2,31E-03	4,6649814	NS	-1,6355486	0,02002159	2,852243
hsa-miR-200a	up	2,03E-03	1,17E-03	1,6974314	NS	1,0075415	0,00265005	1,7102327
hsa-miR-200a*	up	2,03E-03	1,17E-03	1,6974314	NS	1,0075415	0,00265005	1,7102327
hsa-miR-200b	up	7,91E-04	4,39E-03	1,489315	NS	1,1794959	0,00032069	1,7566409
hsa-miR-200b*	up	7,91E-04	4,39E-03	1,489315	NS	1,1794959	0,00032069	1,7566409
hsa-miR-200c	up	4,25E-04	NS	1,2513902	0,01525898	1,3289337	0,00018114	1,6630148
hsa-miR-203	up	NA	NA	NA	NA	NA	NA	NA
hsa-miR-215	up	2,16E-03	NS	1,1535683	0,00630546	1,5746585	0,00122940	1,8164762
hsa-miR-30e	NA	NA	NA	NA	NA	NA	NA	NA
hsa-miR-375	up	NA	NA	NA	NA	NA	NA	NA
hsa-miR-378	NA	3,94E-03	NS	1,7289109	0,04106071	2,0664427	0,00143555	3,5726957
hsa-miR-429	NA	3,05E-03	1,26E-02	1,8412594	NS	1,3115886	0,00125380	2,414975
hsa-miR-96	up	1,48E-02	7,06E-03	1,5354524	NS	-1,1749296	NS	1,3068464

microRNA-Cluster-C	Nishida Epithelia/ Stroma	p(Corr)global	p[LS]vs[MI]	FC[LS]vs[MI]	p[MI]vs[HS]	FC[MI]vs[HS]	p[LS]vs[HS]	FC[LS]vs[HS]
hsa-miR-140-3p	down	NA	NA	NA	NA	NA	NA	NA
hsa-miR-26a	NA	NA	NA	NA	NA	NA	NA	NA
hsa-miR-195	down	4,23E-05	NS	1,1105397	0,00013043	-1,8214186	0,000300415	-1,6401203
hsa-miR-497	down	5,35E-05	NS	1,078612	0,00015121	-1,8256255	0,000246702	-1,6925693
hsa-miR-30a	NA	1,85E-05	NS	1,0554776	0,00012390	-1,7529892	0,000149971	-1,6608493
hsa-miR-143	down	1,79E-05	NS	-1,1755054	0,00019646	-2,0193038	0,000112526	-2,3737025
hsa-miR-145	down	2,31E-05	NS	-1,3832395	0,00128877	-1,7768271	0,000109505	-2,4577773
hsa-miR-376c	down	3,49E-04	NS	1,0573599	0,00062683	-3,6062167	0,000431125	-3,4105856
hsa-let-7c	NA	NA	NA	NA	NA	NA	NA	NA
hsa-miR-193b	NA	1,85E-05	NS	1,0411239	0,00012760	-1,7947067	0,000139761	-1,7238166
hsa-miR-199a-5p	down	1,36E-07	3,90E-02	-1,3574257	0,00012894	-1,9446197	0,000107173	-2,6396768
hsa-miR-214	down	1,07E-05	NS	-1,2850839	0,00074678	-1,5838919	0,000107955	-2,035434
hsa-miR-199a-3p	down	2,50E-08	NS	-1,1921095	0,00011463	-1,9033545	0,000107171	-2,2690067
hsa-miR-130a	down	1,10E-07	3,87E-03	-1,301824	0,00028129	-1,4152312	0,000107171	-1,8423821
hsa-miR-199b-5p	down	2,50E-08	5,26E-03	-1,5091887	0,00014559	-1,8709635	0,000107171	-2,8236368
hsa-miR-100	down	1,05E-06	9,02E-04	-2,5247676	0,00610481	-2,115419	0,000107184	-5,340941
hsa-miR-125b	down	9,73E-10	1,41E-04	-2,1549242	0,00037472	-1,9377195	0,000107171	-4,175638
hsa-let-7i	down	1,79E-07	6,75E-03	-1,2179685	0,00027038	-1,3224956	0,000107172	-1,6107578
hsa-miR-22	down	2,75E-06	6,29E-04	-1,3966582	0,01862598	-1,248951	0,000107233	-1,7443577
hsa-miR-99b	down	7,14E-04	NS	1,0272613	0,00130216	-3,3038135	0,000662063	-3,2161372
hsa-let-7e	down	8,50E-06	NS	1,0766864	0,00011129	-1,6569703	0,00014506	-1,5389534
hsa-miR-125a-5p	down	NA	NA	NA	NA	NA	NA	NA
hsa-miR-23a	NA	NA	NA	NA	NA	NA	NA	NA
hsa-miR-27a	NA	NA	NA	NA	NA	NA	NA	NA
hsa-miR-365	NA	NA	NA	NA	NA	NA	NA	NA
hsa-miR-24-1*	NA	3,81E-04	2,84E-03	1,8917396	0,00029681 4	-2,3719995	NS	-1,2538722
hsa-miR-24	NA	NA	NA	NA	NA	NA	NA	NA

hsa-miR-23b	NA	NA	NA	NA	NA	NA	NA	NA
hsa-miR-27b	NA	NA	NA	NA	NA	NA	NA	NA
