# Supplementary material for: Molecular Classification of Colorectal Cancer by microRNA Profiling: Correlation with the Consensus Molecular Subtypes (CMS) and Validation of miR-30b Targets
Source: Cancers (Basel). 2022 Oct 22;14(21):5175. doi: 10.3390/cancers14215175 (PMC9656292; doi:10.3390/cancers14215175)
Supplement: Supplementary file 1 [file cancers-14-05175-s001.zip › cancers-1966190-supplementary/Supplementary Table S4.pdf]

**Supplementary Table S4. Interactions between miRs and target genes (TALASSO)**

**Interactions between miRs and target genes (TALASSO)**

| Gene | miRNA | pValue |
|---|---|---|
| ENSG00000057593 | hsa-miR-16 | 3,1E-09 |
| ENSG00000164176 | hsa-miR-590-5p | 7,1E-09 |
| ENSG00000100985 | hsa-miR-194* | 3,0E-08 |
| ENSG00000129277 | hsa-miR-183 | 3,7E-07 |
| ENSG00000183098 | hsa-miR-96 | 8,7E-07 |
| ENSG00000149591 | hsa-miR-17* | 9,4E-07 |
| ENSG00000146054 | hsa-miR-361-5p | 1,2E-06 |
| ENSG00000101463 | hsa-miR-194* | 1,5E-06 |
| ENSG00000198467 | hsa-miR-17* | 2,4E-06 |
| ENSG00000130775 | hsa-miR-96 | 2,4E-06 |
| ENSG00000116701 | hsa-miR-378 | 3,1E-06 |
| ENSG00000177469 | hsa-miR-378 | 4,7E-06 |
| ENSG00000176046 | hsa-miR-92a | 5,1E-06 |
| ENSG00000197380 | hsa-miR-378 | 5,7E-06 |
| ENSG00000092758 | hsa-miR-210 | 6,5E-06 |
| ENSG00000111341 | hsa-miR-148a | 8,0E-06 |
| ENSG00000099985 | hsa-let-7g | 8,3E-06 |
| ENSG00000206075 | hsa-miR-378 | 9,3E-06 |
| ENSG00000166033 | hsa-miR-194* | 1,2E-05 |
| ENSG00000162520 | hsa-miR-93 | 1,6E-05 |
| ENSG00000124731 | hsa-miR-96 | 2,9E-05 |
| ENSG00000244414 | hsa-miR-590-5p | 3,0E-05 |
| ENSG00000123500 | hsa-miR-148a | 4,0E-05 |
| ENSG00000079102 | hsa-miR-215 | 4,6E-05 |
| ENSG00000166033 | hsa-miR-93 | 4,6E-05 |
| ENSG00000148926 | hsa-let-7g | 4,8E-05 |
| ENSG00000166866 | hsa-miR-185 | 5,5E-05 |
| ENSG00000140285 | hsa-miR-155 | 5,9E-05 |
| ENSG00000176083 | hsa-miR-194* | 6,5E-05 |
| ENSG00000102265 | hsa-miR-194* | 7,4E-05 |
| ENSG00000122176 | hsa-miR-18a | 9,5E-05 |
| ENSG00000140931 | hsa-miR-425 | 9,9E-05 |
| ENSG00000186198 | hsa-miR-34a | 1,0E-04 |
| ENSG00000162896 | hsa-miR-125b | 1,0E-04 |
| ENSG00000204577 | hsa-miR-378 | 1,0E-04 |
| ENSG00000189334 | hsa-miR-361-5p | 1,0E-04 |
| ENSG00000100292 | hsa-miR-200b | 1,1E-04 |
| ENSG00000169245 | hsa-miR-183 | 1,1E-04 |
| ENSG00000146670 | hsa-miR-214 | 1,4E-04 |
| ENSG00000186340 | hsa-miR-96 | 1,5E-04 |

| | | |
|---|---|---|
| ENSG00000136982 | hsa-miR-26a | 1,6E-04 |
| ENSG00000116741 | hsa-miR-378 | 1,6E-04 |
| ENSG00000107731 | hsa-miR-192 | 1,7E-04 |
| ENSG00000160870 | hsa-miR-18a | 2,0E-04 |
| ENSG00000092758 | hsa-miR-663 | 2,1E-04 |
| ENSG00000106789 | hsa-miR-125b | 2,1E-04 |
| ENSG00000136235 | hsa-miR-200b* | 2,2E-04 |
| ENSG00000182472 | hsa-miR-338-3p | 2,4E-04 |
| ENSG00000167244 | hsa-miR-939 | 2,5E-04 |
| ENSG00000092758 | hsa-miR-30a | 2,5E-04 |
| ENSG00000064205 | hsa-miR-200a* | 2,5E-04 |
| ENSG00000167772 | hsa-miR-192 | 2,6E-04 |
| ENSG00000172215 | hsa-miR-324-5p | 2,8E-04 |
| ENSG00000139174 | hsa-let-7f-1* | 2,9E-04 |
| ENSG00000160188 | hsa-miR-26a | 3,3E-04 |
| ENSG00000122176 | hsa-miR-203 | 3,3E-04 |
| ENSG00000130821 | hsa-miR-19b | 3,4E-04 |
| ENSG00000088836 | hsa-miR-361-5p | 3,5E-04 |
| ENSG00000006074 | hsa-miR-183 | 3,8E-04 |
| ENSG00000163898 | hsa-miR-361-5p | 3,9E-04 |
| ENSG00000129451 | hsa-miR-148a | 4,0E-04 |
| ENSG00000100079 | hsa-miR-125b | 4,0E-04 |
| ENSG00000182326 | hsa-miR-362-5p | 4,1E-04 |
| ENSG00000166147 | hsa-miR-30c | 4,3E-04 |
| ENSG00000211950 | hsa-miR-130b | 4,5E-04 |
| ENSG00000140279 | hsa-miR-125b | 4,5E-04 |
| ENSG00000125869 | hsa-miR-20b | 4,5E-04 |
| ENSG00000131459 | hsa-miR-378 | 4,5E-04 |
| ENSG00000026025 | hsa-miR-93 | 4,6E-04 |
| ENSG00000184254 | hsa-miR-7 | 4,7E-04 |
| ENSG00000130508 | hsa-miR-155 | 4,8E-04 |
| ENSG00000158578 | hsa-miR-378 | 4,8E-04 |
| ENSG00000116701 | hsa-miR-183 | 4,9E-04 |
| ENSG00000143184 | hsa-miR-365 | 5,0E-04 |
| ENSG00000171812 | hsa-miR-196a | 5,2E-04 |
| ENSG00000115523 | hsa-miR-151-5p | 5,5E-04 |
| ENSG00000168461 | hsa-miR-29b | 5,7E-04 |
| ENSG00000175198 | hsa-miR-19a | 6,0E-04 |
| ENSG00000135678 | hsa-miR-196b | 6,4E-04 |
| ENSG00000156804 | hsa-miR-141 | 6,5E-04 |
| ENSG00000120915 | hsa-miR-324-5p | 6,6E-04 |
| ENSG00000169442 | hsa-miR-183 | 6,7E-04 |
| ENSG00000129277 | hsa-miR-378 | 7,2E-04 |
| ENSG00000166523 | hsa-miR-378 | 7,3E-04 |
| ENSG00000089685 | hsa-miR-100 | 7,6E-04 |

| | | |
|---|---|---|
| ENSG00000178075 | hsa-miR-376c | 7,7E-04 |
| ENSG00000120594 | hsa-miR-192 | 7,9E-04 |
| ENSG00000106483 | hsa-miR-96 | 8,1E-04 |
| ENSG00000119938 | hsa-miR-186 | 8,2E-04 |
| ENSG00000112414 | hsa-miR-19a | 8,2E-04 |
| ENSG00000181374 | hsa-miR-96 | 8,5E-04 |
| ENSG00000168502 | hsa-miR-196b | 8,5E-04 |
| ENSG00000184661 | hsa-miR-376c | 8,7E-04 |
| ENSG00000175920 | hsa-miR-221 | 8,9E-04 |
| ENSG00000151623 | hsa-miR-199a-3p | 9,2E-04 |
| ENSG00000143387 | hsa-miR-93 | 9,5E-04 |
| ENSG00000121741 | hsa-miR-15b | 1,0E-03 |
| ENSG00000108244 | hsa-miR-181b | 1,0E-03 |
| ENSG00000154556 | hsa-miR-574-5p | 1,0E-03 |
| ENSG00000090339 | hsa-miR-17* | 1,0E-03 |
| ENSG00000126562 | hsa-miR-142-3p | 1,1E-03 |
| ENSG00000186340 | hsa-miR-29b-1* | 1,1E-03 |
| ENSG00000163347 | hsa-miR-1202 | 1,1E-03 |
| ENSG00000138061 | hsa-miR-19a | 1,1E-03 |
| ENSG00000113140 | hsa-miR-186 | 1,1E-03 |
| ENSG00000172403 | hsa-miR-215 | 1,1E-03 |
| ENSG00000038945 | hsa-miR-1275 | 1,2E-03 |
| ENSG00000182472 | hsa-miR-7 | 1,2E-03 |
| ENSG00000101335 | hsa-miR-92a | 1,2E-03 |
| ENSG00000043462 | hsa-miR-141 | 1,2E-03 |
| ENSG00000172986 | hsa-miR-16 | 1,2E-03 |
| ENSG00000129474 | hsa-miR-148a | 1,2E-03 |
| ENSG00000197766 | hsa-miR-192* | 1,3E-03 |
| ENSG00000154277 | hsa-miR-194* | 1,3E-03 |
| ENSG00000104267 | hsa-miR-1246 | 1,3E-03 |
| ENSG00000169860 | hsa-miR-92a | 1,3E-03 |
| ENSG00000006075 | hsa-miR-194 | 1,3E-03 |
| ENSG00000166816 | hsa-miR-100 | 1,4E-03 |
| ENSG00000138755 | hsa-miR-455-3p | 1,4E-03 |
| ENSG00000163486 | hsa-miR-378 | 1,4E-03 |
| ENSG00000090339 | hsa-miR-221 | 1,4E-03 |
| ENSG00000160181 | hsa-miR-7 | 1,5E-03 |
| ENSG00000168079 | hsa-miR-27a | 1,5E-03 |
| ENSG00000197380 | hsa-miR-1275 | 1,5E-03 |
| ENSG00000111087 | hsa-miR-362-5p | 1,5E-03 |
| ENSG00000171208 | hsa-miR-23b | 1,5E-03 |
| ENSG00000137440 | hsa-miR-25 | 1,5E-03 |
| ENSG00000161647 | hsa-miR-378 | 1,5E-03 |
| ENSG00000142173 | hsa-miR-378 | 1,5E-03 |
| ENSG00000183036 | hsa-miR-574-5p | 1,5E-03 |

| | | |
|---|---|---|
| ENSG00000185915 | hsa-miR-149 | 1,5E-03 |
| ENSG00000164932 | hsa-miR-93 | 1,6E-03 |
| ENSG00000133392 | hsa-miR-625 | 1,6E-03 |
| ENSG00000184661 | hsa-miR-338-3p | 1,6E-03 |
| ENSG00000157227 | hsa-miR-192* | 1,6E-03 |
| ENSG00000163629 | hsa-miR-20b | 1,7E-03 |
| ENSG00000130176 | hsa-miR-194* | 1,7E-03 |
| ENSG00000141756 | hsa-miR-19b | 1,7E-03 |
| ENSG00000122971 | hsa-miR-125b | 1,7E-03 |
| ENSG00000058668 | hsa-miR-1275 | 1,7E-03 |
| ENSG00000163430 | hsa-miR-200b | 1,8E-03 |
| ENSG00000079385 | hsa-miR-34a | 1,8E-03 |
| ENSG00000113140 | hsa-miR-1275 | 1,8E-03 |
| ENSG00000101333 | hsa-miR-939 | 1,8E-03 |
| ENSG00000197635 | hsa-miR-193b | 1,9E-03 |
| ENSG00000164761 | hsa-miR-19a | 1,9E-03 |
| ENSG00000127989 | hsa-miR-140-5p | 1,9E-03 |
| ENSG00000175899 | hsa-miR-362-5p | 1,9E-03 |
| ENSG00000110848 | hsa-miR-183 | 2,0E-03 |
| ENSG00000101335 | hsa-miR-766 | 2,0E-03 |
| ENSG00000205403 | hsa-miR-194 | 2,0E-03 |
| ENSG00000243244 | hsa-miR-532-5p | 2,0E-03 |
| ENSG00000110944 | hsa-miR-20a | 2,0E-03 |
| ENSG00000164109 | hsa-miR-376c | 2,0E-03 |
| ENSG00000166866 | hsa-miR-22 | 2,0E-03 |
| ENSG00000077274 | hsa-let-7e | 2,0E-03 |
| ENSG00000104723 | hsa-miR-155 | 2,0E-03 |
| ENSG00000117650 | hsa-let-7e | 2,0E-03 |
| ENSG00000172201 | hsa-miR-1238 | 2,0E-03 |
| ENSG00000163421 | hsa-miR-497 | 2,0E-03 |
| ENSG00000138755 | hsa-miR-320c | 2,1E-03 |
| ENSG00000203805 | hsa-miR-148a | 2,1E-03 |
| ENSG00000140450 | hsa-miR-20b | 2,1E-03 |
| ENSG00000163734 | hsa-miR-183 | 2,1E-03 |
| ENSG00000163734 | hsa-miR-145 | 2,1E-03 |
| ENSG00000172016 | hsa-miR-342-3p | 2,1E-03 |
| ENSG00000000003 | hsa-miR-625 | 2,1E-03 |
| ENSG00000111729 | hsa-miR-183 | 2,1E-03 |
| ENSG00000101335 | hsa-miR-96 | 2,2E-03 |
| ENSG00000179299 | hsa-miR-940 | 2,2E-03 |
| ENSG00000049323 | hsa-miR-146a | 2,2E-03 |
| ENSG00000107796 | hsa-miR-146a | 2,3E-03 |
| ENSG00000149591 | hsa-miR-1275 | 2,3E-03 |
| ENSG00000162366 | hsa-miR-145 | 2,3E-03 |
| ENSG00000100079 | hsa-miR-200b* | 2,3E-03 |

| | | |
|---|---|---|
| ENSG00000187193 | hsa-miR-192* | 2,3E-03 |
| ENSG00000118271 | hsa-miR-1238 | 2,3E-03 |
| ENSG00000169403 | hsa-miR-196b | 2,3E-03 |
| ENSG00000203706 | hsa-miR-19a | 2,3E-03 |
| ENSG00000112486 | hsa-miR-130a | 2,4E-03 |
| ENSG00000132437 | hsa-miR-22 | 2,5E-03 |
| ENSG00000155760 | hsa-miR-625 | 2,5E-03 |
| ENSG00000117289 | hsa-miR-148a | 2,6E-03 |
| ENSG00000080493 | hsa-miR-1246 | 2,6E-03 |
| ENSG00000183044 | hsa-miR-155 | 2,6E-03 |
| ENSG00000164176 | hsa-miR-425 | 2,6E-03 |
| ENSG00000153234 | hsa-miR-1225-3p | 2,6E-03 |
| ENSG00000166173 | hsa-miR-194* | 2,6E-03 |
| ENSG00000155850 | hsa-let-7c | 2,7E-03 |
| ENSG00000170312 | hsa-miR-23a | 2,7E-03 |
| ENSG00000185269 | hsa-miR-223 | 2,7E-03 |
| ENSG00000135245 | hsa-miR-150 | 2,7E-03 |
| ENSG00000132329 | hsa-miR-378 | 2,7E-03 |
| ENSG00000086289 | hsa-miR-1274a | 2,7E-03 |
| ENSG00000244734 | hsa-miR-378 | 2,7E-03 |
| ENSG00000113916 | hsa-miR-1280 | 2,7E-03 |
| ENSG00000154553 | hsa-miR-146a | 2,8E-03 |
| ENSG00000135245 | hsa-miR-193a-3p | 2,8E-03 |
| ENSG00000080823 | hsa-miR-663 | 2,8E-03 |
| ENSG00000131389 | hsa-miR-200c | 2,8E-03 |
| ENSG00000100292 | hsa-miR-221 | 2,9E-03 |
| ENSG00000168079 | hsa-miR-625 | 2,9E-03 |
| ENSG00000121898 | hsa-miR-19a | 3,0E-03 |
| ENSG00000077157 | hsa-miR-25 | 3,0E-03 |
| ENSG00000182782 | hsa-miR-143 | 3,1E-03 |
| ENSG00000065534 | hsa-miR-320c | 3,1E-03 |
| ENSG00000154839 | hsa-let-7e | 3,1E-03 |
| ENSG00000055118 | hsa-miR-638 | 3,1E-03 |
| ENSG00000077063 | hsa-miR-181b | 3,1E-03 |
| ENSG00000106258 | hsa-let-7i | 3,1E-03 |
| ENSG00000125848 | hsa-miR-1202 | 3,1E-03 |
| ENSG00000146054 | hsa-miR-98 | 3,1E-03 |
| ENSG00000140379 | hsa-miR-27b | 3,2E-03 |
| ENSG00000173404 | hsa-miR-374a | 3,2E-03 |
| ENSG00000166147 | hsa-miR-1274b | 3,3E-03 |
| ENSG00000184661 | hsa-miR-200a | 3,3E-03 |
| ENSG00000145431 | hsa-miR-29b | 3,3E-03 |
| ENSG00000179299 | hsa-miR-185 | 3,3E-03 |
| ENSG00000111087 | hsa-miR-17 | 3,3E-03 |
| ENSG00000180447 | hsa-miR-19a | 3,4E-03 |

| | | |
|---|---|---|
| ENSG00000133110 | hsa-miR-30e | 3,4E-03 |
| ENSG00000167244 | hsa-miR-155 | 3,4E-03 |
| ENSG00000157404 | hsa-miR-338-3p | 3,4E-03 |
| ENSG00000055118 | hsa-miR-1260 | 3,5E-03 |
| ENSG00000198959 | hsa-miR-23b | 3,6E-03 |
| ENSG00000171298 | hsa-miR-200c | 3,7E-03 |
| ENSG00000162366 | hsa-miR-769-5p | 3,8E-03 |
| ENSG00000173404 | hsa-miR-1275 | 3,8E-03 |
| ENSG00000078114 | hsa-miR-140-3p | 3,8E-03 |
| ENSG00000184185 | hsa-miR-185 | 3,9E-03 |
| ENSG00000179299 | hsa-miR-181b | 3,9E-03 |
| ENSG00000167992 | hsa-miR-769-5p | 3,9E-03 |
| ENSG00000155760 | hsa-miR-196a | 4,0E-03 |
| ENSG00000158315 | hsa-miR-1246 | 4,0E-03 |
| ENSG00000119227 | hsa-miR-25 | 4,0E-03 |
| ENSG00000134531 | hsa-miR-148a | 4,0E-03 |
| ENSG00000115295 | hsa-miR-93 | 4,0E-03 |
| ENSG00000163017 | hsa-miR-194 | 4,0E-03 |
| ENSG00000110347 | hsa-miR-26a | 4,1E-03 |
| ENSG00000145824 | hsa-miR-1228 | 4,2E-03 |
| ENSG00000171320 | hsa-miR-23a | 4,2E-03 |
| ENSG00000176387 | hsa-miR-34a | 4,3E-03 |
| ENSG00000129038 | hsa-miR-1274a | 4,3E-03 |
| ENSG00000214021 | hsa-miR-24 | 4,3E-03 |
| ENSG00000168078 | hsa-miR-143 | 4,3E-03 |
| ENSG00000125538 | hsa-miR-590-5p | 4,3E-03 |
| ENSG00000184292 | hsa-miR-186 | 4,3E-03 |
| ENSG00000179299 | hsa-miR-145 | 4,3E-03 |
| ENSG00000178878 | hsa-miR-16 | 4,3E-03 |
| ENSG00000025708 | hsa-miR-574-3p | 4,4E-03 |
| ENSG00000168542 | hsa-miR-186 | 4,4E-03 |
| ENSG00000011465 | hsa-miR-7 | 4,5E-03 |
| ENSG00000091986 | hsa-miR-141 | 4,5E-03 |
| ENSG00000117394 | hsa-miR-148a | 4,5E-03 |
| ENSG00000160255 | hsa-miR-96 | 4,6E-03 |
| ENSG00000043462 | hsa-miR-378 | 4,6E-03 |
| ENSG00000125798 | hsa-let-7b* | 4,7E-03 |
| ENSG00000164530 | hsa-miR-194* | 4,8E-03 |
| ENSG00000140937 | hsa-miR-93 | 4,8E-03 |
| ENSG00000140379 | hsa-miR-98 | 4,8E-03 |
| ENSG00000108244 | hsa-miR-625 | 4,8E-03 |
| ENSG00000066294 | hsa-miR-196b | 4,8E-03 |
| ENSG00000166250 | hsa-miR-130b | 4,8E-03 |
| ENSG00000176083 | hsa-miR-96 | 4,8E-03 |
| ENSG00000115457 | hsa-miR-338-3p | 4,9E-03 |

| | | |
|---|---|---|
| ENSG00000204577 | hsa-miR-361-5p | 4,9E-03 |
| ENSG00000185499 | hsa-miR-769-5p | 5,0E-03 |
| ENSG00000006118 | hsa-miR-29c | 5,0E-03 |
| ENSG00000064655 | hsa-miR-200a* | 5,0E-03 |
| ENSG00000162174 | hsa-miR-125b | 5,0E-03 |
| ENSG00000178462 | hsa-miR-193b | 5,1E-03 |
| ENSG00000077274 | hsa-miR-107 | 5,1E-03 |
| ENSG00000132361 | hsa-let-7e | 5,2E-03 |
| ENSG00000074211 | hsa-let-7i | 5,2E-03 |
| ENSG00000163959 | hsa-miR-625 | 5,3E-03 |
| ENSG00000137752 | hsa-miR-214 | 5,3E-03 |
| ENSG00000162998 | hsa-miR-155 | 5,3E-03 |
| ENSG00000134193 | hsa-miR-378 | 5,4E-03 |
| ENSG00000143882 | hsa-miR-146b-5p | 5,4E-03 |
| ENSG00000164949 | hsa-miR-1275 | 5,4E-03 |
| ENSG00000185479 | hsa-miR-151-3p | 5,4E-03 |
| ENSG00000198700 | hsa-miR-1275 | 5,4E-03 |
| ENSG00000204577 | hsa-miR-20b | 5,5E-03 |
| ENSG00000203706 | hsa-miR-30e* | 5,5E-03 |
| ENSG00000165730 | hsa-miR-27b | 5,6E-03 |
| ENSG00000163328 | hsa-miR-30e | 5,6E-03 |
| ENSG00000102359 | hsa-miR-324-3p | 5,6E-03 |
| ENSG00000145113 | hsa-miR-98 | 5,6E-03 |
| ENSG00000187479 | hsa-miR-574-5p | 5,6E-03 |
| ENSG00000168918 | hsa-miR-21 | 5,7E-03 |
| ENSG00000144802 | hsa-miR-215 | 5,7E-03 |
| ENSG00000171812 | hsa-miR-625 | 5,7E-03 |
| ENSG00000182326 | hsa-miR-30e* | 5,8E-03 |
| ENSG00000134057 | hsa-miR-199a-5p | 5,8E-03 |
| ENSG00000137752 | hsa-miR-98 | 5,8E-03 |
| ENSG00000163735 | hsa-miR-181b | 5,9E-03 |
| ENSG00000158270 | hsa-miR-141 | 5,9E-03 |
| ENSG00000105928 | hsa-miR-194 | 5,9E-03 |
| ENSG00000088992 | hsa-miR-188-5p | 5,9E-03 |
| ENSG00000127585 | hsa-miR-663 | 6,0E-03 |
| ENSG00000165617 | hsa-miR-378 | 6,0E-03 |
| ENSG00000154930 | hsa-miR-21* | 6,1E-03 |
| ENSG00000203857 | hsa-miR-214 | 6,2E-03 |
| ENSG00000105609 | hsa-miR-590-5p | 6,2E-03 |
| ENSG00000137860 | hsa-miR-125b | 6,2E-03 |
| ENSG00000113303 | hsa-miR-1268 | 6,3E-03 |
| ENSG00000175471 | hsa-miR-96 | 6,3E-03 |
| ENSG00000136689 | hsa-let-7g | 6,3E-03 |
| ENSG00000174171 | hsa-miR-22 | 6,4E-03 |
| ENSG00000175745 | hsa-miR-1238 | 6,4E-03 |

| | | |
|---|---|---|
| ENSG00000073849 | hsa-miR-185 | 6,4E-03 |
| ENSG00000132563 | hsa-miR-215 | 6,4E-03 |
| ENSG00000111799 | hsa-miR-19b | 6,4E-03 |
| ENSG00000125337 | hsa-miR-625 | 6,4E-03 |
| ENSG00000112320 | hsa-miR-20a | 6,5E-03 |
| ENSG00000244734 | hsa-miR-374a | 6,5E-03 |
| ENSG00000069424 | hsa-miR-214 | 6,5E-03 |
| ENSG00000057593 | hsa-miR-210 | 6,6E-03 |
| ENSG00000106258 | hsa-miR-769-5p | 6,7E-03 |
| ENSG00000104341 | hsa-miR-625 | 6,7E-03 |
| ENSG00000172403 | hsa-miR-130b | 6,7E-03 |
| ENSG00000169174 | hsa-miR-100 | 6,7E-03 |
| ENSG00000166147 | hsa-let-7d | 6,7E-03 |
| ENSG00000101346 | hsa-miR-1234 | 6,7E-03 |
| ENSG00000136826 | hsa-miR-92a | 6,7E-03 |
| ENSG00000112096 | hsa-miR-30a | 6,8E-03 |
| ENSG00000125730 | hsa-miR-215 | 6,8E-03 |
| ENSG00000104450 | hsa-miR-26b | 6,9E-03 |
| ENSG00000187479 | hsa-miR-191* | 6,9E-03 |
| ENSG00000148926 | hsa-miR-19a | 6,9E-03 |
| ENSG00000126003 | hsa-miR-10a | 6,9E-03 |
| ENSG00000173262 | hsa-miR-18a | 6,9E-03 |
| ENSG00000147883 | hsa-miR-1274a | 7,0E-03 |
| ENSG00000125775 | hsa-miR-663 | 7,0E-03 |
| ENSG00000181458 | hsa-miR-30b | 7,0E-03 |
| ENSG00000166147 | hsa-miR-766 | 7,1E-03 |
| ENSG00000143797 | hsa-miR-1246 | 7,1E-03 |
| ENSG00000167748 | hsa-miR-92a | 7,2E-03 |
| ENSG00000129654 | hsa-miR-141 | 7,2E-03 |
| ENSG00000134193 | hsa-miR-181b | 7,2E-03 |
| ENSG00000171320 | hsa-miR-23b | 7,2E-03 |
| ENSG00000112379 | hsa-miR-18a | 7,2E-03 |
| ENSG00000206538 | hsa-miR-301a | 7,3E-03 |
| ENSG00000148926 | hsa-miR-331-3p | 7,3E-03 |
| ENSG00000124212 | hsa-miR-186 | 7,3E-03 |
| ENSG00000142677 | hsa-let-7c | 7,3E-03 |
| ENSG00000176842 | hsa-miR-196a | 7,3E-03 |
| ENSG00000177459 | hsa-miR-146a | 7,4E-03 |
| ENSG00000121741 | hsa-miR-21 | 7,4E-03 |
| ENSG00000125848 | hsa-miR-15a | 7,4E-03 |
| ENSG00000183018 | hsa-miR-27a | 7,4E-03 |
| ENSG00000101335 | hsa-miR-142-3p | 7,4E-03 |
| ENSG00000107159 | hsa-miR-23a | 7,5E-03 |
| ENSG00000090920 | hsa-miR-23a | 7,5E-03 |
| ENSG00000115363 | hsa-miR-142-3p | 7,5E-03 |

| | | |
|---|---|---|
| ENSG00000108375 | hsa-let-7a | 7,5E-03 |
| ENSG00000108700 | hsa-miR-148a | 7,6E-03 |
| ENSG00000077782 | hsa-miR-93 | 7,6E-03 |
| ENSG00000140284 | hsa-miR-199a-3p | 7,6E-03 |
| ENSG00000145569 | hsa-miR-155 | 7,6E-03 |
| ENSG00000145147 | hsa-miR-200c | 7,6E-03 |
| ENSG00000152377 | hsa-miR-361-5p | 7,6E-03 |
| ENSG00000101346 | hsa-miR-34a | 7,7E-03 |
| ENSG00000148735 | hsa-miR-18a | 7,7E-03 |
| ENSG00000137573 | hsa-miR-1260 | 7,7E-03 |
| ENSG00000129514 | hsa-miR-142-3p | 7,7E-03 |
| ENSG00000089177 | hsa-miR-15b | 7,7E-03 |
| ENSG00000104290 | hsa-miR-939 | 7,8E-03 |
| ENSG00000143882 | hsa-miR-146a | 7,8E-03 |
| ENSG00000102837 | hsa-miR-151-5p | 7,8E-03 |
| ENSG00000147883 | hsa-miR-148a | 7,8E-03 |
| ENSG00000184185 | hsa-miR-320d | 7,8E-03 |
| ENSG00000148082 | hsa-miR-324-3p | 7,8E-03 |
| ENSG00000105063 | hsa-miR-96 | 7,9E-03 |
| ENSG00000160255 | hsa-miR-29b | 7,9E-03 |
| ENSG00000167992 | hsa-miR-146a | 7,9E-03 |
| ENSG00000185479 | hsa-miR-148a | 7,9E-03 |
| ENSG00000176194 | hsa-miR-425 | 7,9E-03 |
| ENSG00000125869 | hsa-miR-93 | 7,9E-03 |
| ENSG00000110079 | hsa-miR-20a | 7,9E-03 |
| ENSG00000159674 | hsa-miR-378 | 7,9E-03 |
| ENSG00000175463 | hsa-miR-96 | 7,9E-03 |
| ENSG00000086289 | hsa-miR-200b | 8,0E-03 |
| ENSG00000171234 | hsa-miR-199a-3p | 8,0E-03 |
| ENSG00000103485 | hsa-miR-15b | 8,1E-03 |
| ENSG00000140284 | hsa-miR-200a | 8,1E-03 |
| ENSG00000163739 | hsa-miR-181b | 8,1E-03 |
| ENSG00000145649 | hsa-miR-27b | 8,1E-03 |
| ENSG00000122971 | hsa-miR-197 | 8,2E-03 |
| ENSG00000121413 | hsa-miR-142-3p | 8,2E-03 |
| ENSG00000166923 | hsa-miR-1260 | 8,2E-03 |
| ENSG00000149591 | hsa-miR-185 | 8,2E-03 |
| ENSG00000136193 | hsa-miR-181b | 8,3E-03 |
| ENSG00000197614 | hsa-miR-20b | 8,3E-03 |
| ENSG00000138193 | hsa-miR-20a | 8,4E-03 |
| ENSG00000158315 | hsa-miR-25 | 8,4E-03 |
| ENSG00000120068 | hsa-miR-149 | 8,4E-03 |
| ENSG00000139926 | hsa-miR-20a | 8,4E-03 |
| ENSG00000112742 | hsa-miR-26a | 8,4E-03 |
| ENSG00000177469 | hsa-miR-93 | 8,5E-03 |

| | | |
|---|---|---|
| ENSG00000006747 | hsa-miR-18a | 8,5E-03 |
| ENSG00000143546 | hsa-miR-98 | 8,5E-03 |
| ENSG00000041982 | hsa-miR-19b | 8,6E-03 |
| ENSG00000173404 | hsa-miR-221 | 8,6E-03 |
| ENSG00000158859 | hsa-miR-497 | 8,6E-03 |
| ENSG00000154175 | hsa-miR-374a | 8,7E-03 |
| ENSG00000130176 | hsa-miR-625 | 8,7E-03 |
| ENSG00000106819 | hsa-miR-200c | 8,9E-03 |
| ENSG00000112818 | hsa-miR-214 | 8,9E-03 |
| ENSG00000077942 | hsa-miR-183 | 8,9E-03 |
| ENSG00000107159 | hsa-miR-15a | 9,0E-03 |
| ENSG00000140937 | hsa-miR-200b | 9,1E-03 |
| ENSG00000170345 | hsa-miR-338-3p | 9,1E-03 |
| ENSG00000152518 | hsa-miR-375 | 9,2E-03 |
| ENSG00000100234 | hsa-miR-940 | 9,2E-03 |
| ENSG00000078098 | hsa-miR-429 | 9,2E-03 |
| ENSG00000000971 | hsa-miR-590-5p | 9,2E-03 |
| ENSG00000170801 | hsa-miR-183 | 9,2E-03 |
| ENSG00000152518 | hsa-miR-29b-1* | 9,2E-03 |
| ENSG00000080546 | hsa-miR-766 | 9,3E-03 |
| ENSG00000077157 | hsa-let-7d | 9,3E-03 |
| ENSG00000182326 | hsa-miR-210 | 9,3E-03 |
| ENSG00000158467 | hsa-miR-195 | 9,4E-03 |
| ENSG00000183098 | hsa-miR-17 | 9,4E-03 |
| ENSG00000137501 | hsa-miR-142-3p | 9,4E-03 |
| ENSG00000152503 | hsa-miR-195 | 9,4E-03 |
| ENSG00000165617 | hsa-miR-140-5p | 9,4E-03 |
| ENSG00000129451 | hsa-let-7g | 9,5E-03 |
| ENSG00000138207 | hsa-miR-886-3p | 9,5E-03 |
| ENSG00000084636 | hsa-miR-194* | 9,6E-03 |
| ENSG00000017427 | hsa-miR-194* | 9,6E-03 |
| ENSG00000065833 | hsa-miR-92a | 9,6E-03 |
| ENSG00000108700 | hsa-miR-92a | 9,7E-03 |
| ENSG00000108176 | hsa-miR-1246 | 9,7E-03 |
| ENSG00000175084 | hsa-miR-1260 | 9,7E-03 |
| ENSG00000086589 | hsa-miR-590-5p | 9,7E-03 |
| ENSG00000101049 | hsa-miR-140-5p | 9,7E-03 |
| ENSG00000166391 | hsa-miR-199a-5p | 9,7E-03 |
| ENSG00000172016 | hsa-miR-23a | 9,7E-03 |
| ENSG00000176907 | hsa-miR-199a-3p | 9,8E-03 |
| ENSG00000140931 | hsa-miR-16 | 1,0E-02 |
| ENSG00000176907 | hsa-miR-494 | 1,0E-02 |
| ENSG00000006118 | hsa-miR-29a | 1,0E-02 |
| ENSG00000153234 | hsa-miR-494 | 1,0E-02 |
| ENSG00000189221 | hsa-miR-455-3p | 1,0E-02 |

| | | |
|---|---|---|
| ENSG00000006075 | hsa-miR-24-1* | 1,0E-02 |
| ENSG00000204262 | hsa-miR-30c | 1,0E-02 |
| ENSG00000143333 | hsa-miR-30e | 1,0E-02 |
| ENSG00000004399 | hsa-miR-221 | 1,0E-02 |
| ENSG00000105976 | hsa-miR-130a | 1,0E-02 |
| ENSG00000110944 | hsa-miR-362-5p | 1,0E-02 |
| ENSG00000116711 | hsa-miR-361-5p | 1,0E-02 |
| ENSG00000147394 | hsa-miR-532-5p | 1,0E-02 |
| ENSG00000197272 | hsa-miR-451 | 1,0E-02 |
| ENSG00000123096 | hsa-miR-141 | 1,0E-02 |
| ENSG00000163683 | hsa-miR-19a | 1,0E-02 |
| ENSG00000136235 | hsa-miR-660 | 1,0E-02 |
| ENSG00000203706 | hsa-miR-30d | 1,0E-02 |
| ENSG00000211626 | hsa-miR-200b* | 1,0E-02 |
| ENSG00000167757 | hsa-miR-29b | 1,0E-02 |
| ENSG00000159261 | hsa-miR-455-3p | 1,0E-02 |
| ENSG00000239839 | hsa-miR-194 | 1,0E-02 |
| ENSG00000110013 | hsa-miR-1246 | 1,1E-02 |
| ENSG00000169903 | hsa-miR-221 | 1,1E-02 |
| ENSG00000186198 | hsa-miR-181b | 1,1E-02 |
| ENSG00000154269 | hsa-miR-146b-5p | 1,1E-02 |
| ENSG00000138821 | hsa-miR-181b | 1,1E-02 |
| ENSG00000128849 | hsa-let-7f | 1,1E-02 |
| ENSG00000104142 | hsa-miR-196a | 1,1E-02 |
| ENSG00000112320 | hsa-miR-25 | 1,1E-02 |
| ENSG00000204335 | hsa-miR-7 | 1,1E-02 |
| ENSG00000149591 | hsa-miR-196a | 1,1E-02 |
| ENSG00000118407 | hsa-miR-20b | 1,1E-02 |
| ENSG00000120738 | hsa-miR-200b | 1,1E-02 |
| ENSG00000179299 | hsa-miR-15a | 1,1E-02 |
| ENSG00000139926 | hsa-miR-200c | 1,1E-02 |
| ENSG00000196154 | hsa-miR-92a | 1,1E-02 |
| ENSG00000179603 | hsa-miR-24 | 1,1E-02 |
| ENSG00000203805 | hsa-miR-30d | 1,1E-02 |
| ENSG00000198947 | hsa-miR-155 | 1,1E-02 |
| ENSG00000163683 | hsa-miR-27a | 1,1E-02 |
| ENSG00000151726 | hsa-miR-196b | 1,1E-02 |
| ENSG00000152661 | hsa-miR-181b | 1,1E-02 |
| ENSG00000145936 | hsa-miR-146a | 1,1E-02 |
| ENSG00000113140 | hsa-miR-625 | 1,1E-02 |
| ENSG00000130988 | hsa-miR-155 | 1,1E-02 |
| ENSG00000131746 | hsa-miR-1207-5p | 1,1E-02 |
| ENSG00000163430 | hsa-miR-183 | 1,1E-02 |
| ENSG00000099984 | hsa-miR-324-3p | 1,1E-02 |
| ENSG00000124107 | hsa-miR-130b | 1,1E-02 |

| | | |
|---|---|---|
| ENSG00000184347 | hsa-miR-200b | 1,1E-02 |
| ENSG00000006118 | hsa-miR-194* | 1,1E-02 |
| ENSG00000166825 | hsa-miR-151-3p | 1,1E-02 |
| ENSG00000131389 | hsa-miR-30b | 1,1E-02 |
| ENSG00000156127 | hsa-miR-27a | 1,1E-02 |
| ENSG00000106624 | hsa-miR-93 | 1,2E-02 |
| ENSG00000126003 | hsa-miR-22 | 1,2E-02 |
| ENSG00000123358 | hsa-miR-101 | 1,2E-02 |
| ENSG00000050405 | hsa-miR-125b | 1,2E-02 |
| ENSG00000213886 | hsa-miR-200b* | 1,2E-02 |
| ENSG00000102471 | hsa-miR-130b | 1,2E-02 |
| ENSG00000168785 | hsa-miR-16 | 1,2E-02 |
| ENSG00000053918 | hsa-miR-331-3p | 1,2E-02 |
| ENSG00000163909 | hsa-miR-766 | 1,2E-02 |
| ENSG00000169271 | hsa-miR-181b | 1,2E-02 |
| ENSG00000162078 | hsa-miR-324-3p | 1,2E-02 |
| ENSG00000170745 | hsa-miR-18a | 1,2E-02 |
| ENSG00000055118 | hsa-miR-155 | 1,2E-02 |
| ENSG00000152661 | hsa-miR-30c | 1,2E-02 |
| ENSG00000185275 | hsa-miR-146b-5p | 1,2E-02 |
| ENSG00000143001 | hsa-miR-374a | 1,2E-02 |
| ENSG00000181804 | hsa-let-7d | 1,2E-02 |
| ENSG00000110077 | hsa-miR-141 | 1,2E-02 |
| ENSG00000166165 | hsa-miR-638 | 1,2E-02 |
| ENSG00000019505 | hsa-miR-221 | 1,2E-02 |
| ENSG00000175198 | hsa-miR-338-3p | 1,2E-02 |
| ENSG00000174171 | hsa-miR-107 | 1,2E-02 |
| ENSG00000169474 | hsa-miR-574-5p | 1,2E-02 |
| ENSG00000026751 | hsa-miR-200a* | 1,2E-02 |
| ENSG00000127585 | hsa-let-7f | 1,2E-02 |
| ENSG00000148926 | hsa-miR-20a | 1,2E-02 |
| ENSG00000189221 | hsa-miR-34a | 1,2E-02 |
| ENSG00000205364 | hsa-miR-96 | 1,2E-02 |
| ENSG00000181061 | hsa-miR-19b | 1,2E-02 |
| ENSG00000163083 | hsa-miR-210 | 1,3E-02 |
| ENSG00000132437 | hsa-miR-24-1* | 1,3E-02 |
| ENSG00000139329 | hsa-miR-146a | 1,3E-02 |
| ENSG00000120068 | hsa-miR-30b | 1,3E-02 |
| ENSG00000094804 | hsa-miR-26a | 1,3E-02 |
| ENSG00000139174 | hsa-miR-532-5p | 1,3E-02 |
| ENSG00000110013 | hsa-miR-30b | 1,3E-02 |
| ENSG00000091986 | hsa-miR-96 | 1,3E-02 |
| ENSG00000115386 | hsa-miR-199a-3p | 1,3E-02 |
| ENSG00000186352 | hsa-miR-338-3p | 1,3E-02 |
| ENSG00000074211 | hsa-miR-15a | 1,3E-02 |

| | | |
|---|---|---|
| ENSG00000123500 | hsa-miR-106b | 1,3E-02 |
| ENSG00000066294 | hsa-miR-200c | 1,3E-02 |
| ENSG00000145423 | hsa-miR-20b | 1,3E-02 |
| ENSG00000102837 | hsa-miR-22 | 1,3E-02 |
| ENSG00000178878 | hsa-miR-194 | 1,3E-02 |
| ENSG00000135226 | hsa-miR-7 | 1,3E-02 |
| ENSG00000017427 | hsa-miR-331-3p | 1,3E-02 |
| ENSG00000143797 | hsa-miR-18a | 1,3E-02 |
| ENSG00000026025 | hsa-miR-17* | 1,3E-02 |
| ENSG00000163909 | hsa-miR-19b | 1,3E-02 |
| ENSG00000110080 | hsa-miR-92a | 1,3E-02 |
| ENSG00000139926 | hsa-miR-183 | 1,3E-02 |
| ENSG00000074527 | hsa-miR-320c | 1,3E-02 |
| ENSG00000026025 | hsa-miR-30c | 1,3E-02 |
| ENSG00000146592 | hsa-miR-146a | 1,3E-02 |
| ENSG00000131203 | hsa-miR-148b | 1,3E-02 |
| ENSG00000198729 | hsa-miR-146b-5p | 1,3E-02 |
| ENSG00000143882 | hsa-miR-183 | 1,3E-02 |
| ENSG00000068078 | hsa-miR-1207-5p | 1,3E-02 |
| ENSG00000165828 | hsa-miR-34a | 1,3E-02 |
| ENSG00000147027 | hsa-miR-20b | 1,3E-02 |
| ENSG00000159231 | hsa-miR-92a | 1,3E-02 |
| ENSG00000121211 | hsa-miR-30c | 1,3E-02 |
| ENSG00000111087 | hsa-miR-200c | 1,3E-02 |
| ENSG00000121741 | hsa-let-7b* | 1,3E-02 |
| ENSG00000100448 | hsa-miR-200c | 1,3E-02 |
| ENSG00000109472 | hsa-miR-142-3p | 1,3E-02 |
| ENSG00000147394 | hsa-miR-20a | 1,4E-02 |
| ENSG00000197635 | hsa-miR-196b | 1,4E-02 |
| ENSG00000104783 | hsa-miR-296-5p | 1,4E-02 |
| ENSG00000138821 | hsa-miR-145 | 1,4E-02 |
| ENSG00000120068 | hsa-miR-1249 | 1,4E-02 |
| ENSG00000176153 | hsa-miR-143 | 1,4E-02 |
| ENSG00000143333 | hsa-let-7f | 1,4E-02 |
| ENSG00000166147 | hsa-miR-590-5p | 1,4E-02 |
| ENSG00000165757 | hsa-miR-200b | 1,4E-02 |
| ENSG00000066294 | hsa-miR-301a | 1,4E-02 |
| ENSG00000134443 | hsa-miR-196a | 1,4E-02 |
| ENSG00000110848 | hsa-miR-148a | 1,4E-02 |
| ENSG00000163430 | hsa-miR-378 | 1,4E-02 |
| ENSG00000125968 | hsa-miR-663 | 1,4E-02 |
| ENSG00000053747 | hsa-miR-7 | 1,4E-02 |
| ENSG00000135245 | hsa-miR-181b | 1,4E-02 |
| ENSG00000224557 | hsa-miR-29b | 1,4E-02 |
| ENSG00000148926 | hsa-miR-181a | 1,4E-02 |

| | | |
|---|---|---|
| ENSG00000163520 | hsa-miR-7 | 1,4E-02 |
| ENSG00000073756 | hsa-miR-155 | 1,4E-02 |
| ENSG00000123838 | hsa-miR-34a | 1,4E-02 |
| ENSG00000117394 | hsa-miR-1249 | 1,4E-02 |
| ENSG00000132437 | hsa-miR-34a | 1,4E-02 |
| ENSG00000137699 | hsa-miR-21 | 1,4E-02 |
| ENSG00000102409 | hsa-miR-183 | 1,4E-02 |
| ENSG00000166741 | hsa-miR-29b | 1,5E-02 |
| ENSG00000198467 | hsa-miR-1275 | 1,5E-02 |
| ENSG00000111087 | hsa-miR-766 | 1,5E-02 |
| ENSG00000079689 | hsa-miR-425 | 1,5E-02 |
| ENSG00000130821 | hsa-let-7e | 1,5E-02 |
| ENSG00000161647 | hsa-miR-1225-3p | 1,5E-02 |
| ENSG00000175426 | hsa-miR-142-3p | 1,5E-02 |
| ENSG00000136997 | hsa-miR-22 | 1,5E-02 |
| ENSG00000119938 | hsa-miR-19a | 1,5E-02 |
| ENSG00000101333 | hsa-miR-155 | 1,5E-02 |
| ENSG00000117394 | hsa-miR-375 | 1,5E-02 |
| ENSG00000165092 | hsa-miR-17* | 1,5E-02 |
| ENSG00000105976 | hsa-miR-155 | 1,5E-02 |
| ENSG00000176945 | hsa-miR-342-3p | 1,5E-02 |
| ENSG00000119121 | hsa-let-7c | 1,5E-02 |
| ENSG00000178828 | hsa-miR-130a | 1,5E-02 |
| ENSG00000139211 | hsa-miR-142-3p | 1,5E-02 |
| ENSG00000026025 | hsa-miR-96 | 1,5E-02 |
| ENSG00000163083 | hsa-miR-16 | 1,5E-02 |
| ENSG00000115415 | hsa-miR-221 | 1,5E-02 |
| ENSG00000131711 | hsa-miR-200c | 1,5E-02 |
| ENSG00000026036 | hsa-miR-324-3p | 1,5E-02 |
| ENSG00000173262 | hsa-miR-320d | 1,5E-02 |
| ENSG00000106538 | hsa-miR-29b | 1,5E-02 |
| ENSG00000116785 | hsa-miR-361-5p | 1,5E-02 |
| ENSG00000104290 | hsa-miR-23b | 1,5E-02 |
| ENSG00000154734 | hsa-miR-1274b | 1,5E-02 |
| ENSG00000163486 | hsa-miR-20a | 1,5E-02 |
| ENSG00000198729 | hsa-miR-362-5p | 1,5E-02 |
| ENSG00000134363 | hsa-miR-1275 | 1,5E-02 |
| ENSG00000124225 | hsa-miR-96 | 1,5E-02 |
| ENSG00000066294 | hsa-miR-574-5p | 1,5E-02 |
| ENSG00000136231 | hsa-miR-30e | 1,5E-02 |
| ENSG00000123243 | hsa-miR-766 | 1,6E-02 |
| ENSG00000084636 | hsa-miR-19a | 1,6E-02 |
| ENSG00000137440 | hsa-miR-100 | 1,6E-02 |
| ENSG00000122641 | hsa-miR-150 | 1,6E-02 |
| ENSG00000138755 | hsa-miR-30e | 1,6E-02 |

| | | |
|---|---|---|
| ENSG00000117525 | hsa-miR-199a-3p | 1,6E-02 |
| ENSG00000206075 | hsa-miR-21 | 1,6E-02 |
| ENSG00000104432 | hsa-miR-362-5p | 1,6E-02 |
| ENSG00000122641 | hsa-miR-574-5p | 1,6E-02 |
| ENSG00000197635 | hsa-miR-148b | 1,6E-02 |
| ENSG00000146592 | hsa-miR-130b | 1,6E-02 |
| ENSG00000004399 | hsa-let-7f | 1,6E-02 |
| ENSG00000135447 | hsa-miR-29b | 1,6E-02 |
| ENSG00000004399 | hsa-miR-375 | 1,6E-02 |
| ENSG00000169439 | hsa-miR-150 | 1,6E-02 |
| ENSG00000180447 | hsa-miR-93 | 1,6E-02 |
| ENSG00000135046 | hsa-miR-494 | 1,6E-02 |
| ENSG00000137573 | hsa-miR-200b | 1,6E-02 |
| ENSG00000136244 | hsa-miR-155 | 1,6E-02 |
| ENSG00000151623 | hsa-miR-18a | 1,6E-02 |
| ENSG00000070526 | hsa-miR-26b | 1,6E-02 |
| ENSG00000166922 | hsa-miR-18a | 1,6E-02 |
| ENSG00000154721 | hsa-miR-192* | 1,6E-02 |
| ENSG00000132749 | hsa-miR-146b-5p | 1,6E-02 |
| ENSG00000124006 | hsa-miR-362-5p | 1,6E-02 |
| ENSG00000157601 | hsa-miR-574-3p | 1,6E-02 |
| ENSG00000134363 | hsa-miR-141 | 1,6E-02 |
| ENSG00000153283 | hsa-miR-96 | 1,6E-02 |
| ENSG00000100453 | hsa-miR-199a-5p | 1,6E-02 |
| ENSG00000184254 | hsa-miR-200c | 1,6E-02 |
| ENSG00000133134 | hsa-miR-196a | 1,6E-02 |
| ENSG00000164692 | hsa-miR-19a | 1,6E-02 |
| ENSG00000136826 | hsa-miR-34a | 1,6E-02 |
| ENSG00000135111 | hsa-miR-26a | 1,6E-02 |
| ENSG00000110079 | hsa-miR-203 | 1,7E-02 |
| ENSG00000242110 | hsa-miR-181b | 1,7E-02 |
| ENSG00000163735 | hsa-miR-141 | 1,7E-02 |
| ENSG00000173404 | hsa-miR-766 | 1,7E-02 |
| ENSG00000172164 | hsa-miR-338-3p | 1,7E-02 |
| ENSG00000198467 | hsa-miR-96 | 1,7E-02 |
| ENSG00000161647 | hsa-miR-181b | 1,7E-02 |
| ENSG00000142871 | hsa-miR-429 | 1,7E-02 |
| ENSG00000169282 | hsa-miR-196a | 1,7E-02 |
| ENSG00000049323 | hsa-miR-10a | 1,7E-02 |
| ENSG00000103707 | hsa-miR-196b | 1,7E-02 |
| ENSG00000166831 | hsa-miR-92a | 1,7E-02 |
| ENSG00000154175 | hsa-miR-194 | 1,7E-02 |
| ENSG00000143954 | hsa-miR-143 | 1,7E-02 |
| ENSG00000076864 | hsa-miR-27a | 1,7E-02 |
| ENSG00000112486 | hsa-miR-130b | 1,7E-02 |

| | | |
|---|---|---|
| ENSG00000132386 | hsa-miR-494 | 1,7E-02 |
| ENSG00000120708 | hsa-miR-200c | 1,7E-02 |
| ENSG00000085741 | hsa-miR-21* | 1,7E-02 |
| ENSG00000179299 | hsa-miR-23a | 1,7E-02 |
| ENSG00000038945 | hsa-miR-590-5p | 1,7E-02 |
| ENSG00000080546 | hsa-miR-15b | 1,7E-02 |
| ENSG00000112379 | hsa-miR-494 | 1,7E-02 |
| ENSG00000172986 | hsa-miR-29b | 1,7E-02 |
| ENSG00000124882 | hsa-miR-215 | 1,7E-02 |
| ENSG00000130513 | hsa-miR-151-5p | 1,7E-02 |
| ENSG00000119125 | hsa-miR-130a | 1,7E-02 |
| ENSG00000131459 | hsa-miR-93 | 1,7E-02 |
| ENSG00000138615 | hsa-miR-203 | 1,7E-02 |
| ENSG00000167286 | hsa-miR-151-5p | 1,7E-02 |
| ENSG00000066294 | hsa-miR-10a | 1,7E-02 |
| ENSG00000106025 | hsa-miR-34a | 1,7E-02 |
| ENSG00000135046 | hsa-miR-590-5p | 1,7E-02 |
| ENSG00000125869 | hsa-miR-142-3p | 1,8E-02 |
| ENSG00000139211 | hsa-miR-96 | 1,8E-02 |
| ENSG00000163618 | hsa-miR-140-5p | 1,8E-02 |
| ENSG00000121898 | hsa-let-7f | 1,8E-02 |
| ENSG00000095739 | hsa-miR-7 | 1,8E-02 |
| ENSG00000158201 | hsa-miR-125b | 1,8E-02 |
| ENSG00000182580 | hsa-miR-1275 | 1,8E-02 |
| ENSG00000125968 | hsa-miR-29c | 1,8E-02 |
| ENSG00000135083 | hsa-miR-150 | 1,8E-02 |
| ENSG00000179299 | hsa-miR-19a | 1,8E-02 |
| ENSG00000141756 | hsa-let-7d | 1,8E-02 |
| ENSG00000079102 | hsa-miR-17 | 1,8E-02 |
| ENSG00000178828 | hsa-miR-26b | 1,8E-02 |
| ENSG00000152583 | hsa-miR-148a | 1,8E-02 |
| ENSG00000086696 | hsa-miR-223 | 1,8E-02 |
| ENSG00000203943 | hsa-miR-188-5p | 1,8E-02 |
| ENSG00000119508 | hsa-miR-19b | 1,8E-02 |
| ENSG00000185090 | hsa-let-7a | 1,8E-02 |
| ENSG00000116661 | hsa-miR-181b | 1,9E-02 |
| ENSG00000078596 | hsa-miR-103 | 1,9E-02 |
| ENSG00000178075 | hsa-miR-1260 | 1,9E-02 |
| ENSG00000118523 | hsa-miR-19b | 1,9E-02 |
| ENSG00000183098 | hsa-miR-107 | 1,9E-02 |
| ENSG00000166866 | hsa-miR-221 | 1,9E-02 |
| ENSG00000142875 | hsa-miR-338-3p | 1,9E-02 |
| ENSG00000168903 | hsa-miR-374a | 1,9E-02 |
| ENSG00000168502 | hsa-miR-196a | 1,9E-02 |
| ENSG00000168079 | hsa-miR-17 | 1,9E-02 |

| | | |
|---|---|---|
| ENSG00000163347 | hsa-miR-186 | 1,9E-02 |
| ENSG00000011465 | hsa-miR-146a | 1,9E-02 |
| ENSG00000136244 | hsa-miR-203 | 1,9E-02 |
| ENSG00000077782 | hsa-miR-30c | 1,9E-02 |
| ENSG00000145649 | hsa-miR-19b | 1,9E-02 |
| ENSG00000224557 | hsa-miR-769-5p | 1,9E-02 |
| ENSG00000122257 | hsa-miR-29b-1* | 1,9E-02 |
| ENSG00000122694 | hsa-miR-454 | 1,9E-02 |
| ENSG00000106688 | hsa-miR-199b-5p | 1,9E-02 |
| ENSG00000101938 | hsa-miR-425 | 1,9E-02 |
| ENSG00000130508 | hsa-miR-93 | 1,9E-02 |
| ENSG00000182580 | hsa-miR-454 | 1,9E-02 |
| ENSG00000135069 | hsa-miR-16 | 1,9E-02 |
| ENSG00000244414 | hsa-miR-29b-1* | 1,9E-02 |
| ENSG00000107562 | hsa-miR-103 | 1,9E-02 |
| ENSG00000213886 | hsa-miR-375 | 1,9E-02 |
| ENSG00000088992 | hsa-miR-766 | 1,9E-02 |
| ENSG00000100078 | hsa-miR-301a | 1,9E-02 |
| ENSG00000050405 | hsa-miR-20a | 1,9E-02 |
| ENSG00000125775 | hsa-miR-34a | 1,9E-02 |
| ENSG00000127585 | hsa-miR-324-3p | 1,9E-02 |
| ENSG00000163347 | hsa-miR-301a | 1,9E-02 |
| ENSG00000181061 | hsa-miR-1308 | 1,9E-02 |
| ENSG00000113140 | hsa-miR-29b | 1,9E-02 |
| ENSG00000171004 | hsa-miR-30c | 2,0E-02 |
| ENSG00000164920 | hsa-let-7g | 2,0E-02 |
| ENSG00000102678 | hsa-miR-140-5p | 2,0E-02 |
| ENSG00000138795 | hsa-miR-93 | 2,0E-02 |
| ENSG00000115380 | hsa-miR-7 | 2,0E-02 |
| ENSG00000145824 | hsa-miR-455-3p | 2,0E-02 |
| ENSG00000122641 | hsa-miR-93 | 2,0E-02 |
| ENSG00000162772 | hsa-miR-23a | 2,0E-02 |
| ENSG00000168646 | hsa-miR-625 | 2,0E-02 |
| ENSG00000157315 | hsa-miR-660 | 2,0E-02 |
| ENSG00000101000 | hsa-miR-10b | 2,0E-02 |
| ENSG00000163898 | hsa-miR-125b | 2,0E-02 |
| ENSG00000100078 | hsa-miR-92a | 2,0E-02 |
| ENSG00000187758 | hsa-miR-625 | 2,0E-02 |
| ENSG00000087245 | hsa-miR-20a | 2,0E-02 |
| ENSG00000124006 | hsa-miR-29b | 2,0E-02 |
| ENSG00000111404 | hsa-miR-574-5p | 2,0E-02 |
| ENSG00000099984 | hsa-miR-141 | 2,0E-02 |
| ENSG00000206075 | hsa-miR-25 | 2,0E-02 |
| ENSG00000180801 | hsa-miR-26b | 2,0E-02 |
| ENSG00000120885 | hsa-miR-196a | 2,0E-02 |

| | | |
|---|---|---|
| ENSG00000083720 | hsa-miR-20a | 2,0E-02 |
| ENSG00000102445 | hsa-miR-10b | 2,0E-02 |
| ENSG00000125845 | hsa-miR-181b | 2,0E-02 |
| ENSG00000182326 | hsa-miR-130b | 2,0E-02 |
| ENSG00000117650 | hsa-let-7g | 2,0E-02 |
| ENSG00000135750 | hsa-miR-19a | 2,0E-02 |
| ENSG00000181061 | hsa-miR-199a-5p | 2,0E-02 |
| ENSG00000152952 | hsa-miR-96 | 2,0E-02 |
| ENSG00000196616 | hsa-miR-625 | 2,1E-02 |
| ENSG00000161551 | hsa-miR-196a | 2,1E-02 |
| ENSG00000146592 | hsa-miR-20a | 2,1E-02 |
| ENSG00000106624 | hsa-miR-30e* | 2,1E-02 |
| ENSG00000102265 | hsa-miR-200b | 2,1E-02 |
| ENSG00000122861 | hsa-miR-19b | 2,1E-02 |
| ENSG00000175538 | hsa-miR-769-5p | 2,1E-02 |
| ENSG00000166173 | hsa-miR-425* | 2,1E-02 |
| ENSG00000211965 | hsa-miR-130b | 2,1E-02 |
| ENSG00000010030 | hsa-miR-30c | 2,1E-02 |
| ENSG00000130821 | hsa-let-7g | 2,1E-02 |
| ENSG00000006210 | hsa-miR-183 | 2,1E-02 |
| ENSG00000185156 | hsa-miR-20b | 2,1E-02 |
| ENSG00000178462 | hsa-miR-100 | 2,1E-02 |
| ENSG00000198729 | hsa-miR-10b | 2,1E-02 |
| ENSG00000111341 | hsa-miR-107 | 2,1E-02 |
| ENSG00000140937 | hsa-miR-19a | 2,1E-02 |
| ENSG00000006606 | hsa-miR-194* | 2,1E-02 |
| ENSG00000129538 | hsa-let-7g | 2,1E-02 |
| ENSG00000136999 | hsa-miR-25 | 2,1E-02 |
| ENSG00000129451 | hsa-miR-1207-5p | 2,2E-02 |
| ENSG00000172156 | hsa-miR-625 | 2,2E-02 |
| ENSG00000078399 | hsa-miR-26a | 2,2E-02 |
| ENSG00000153936 | hsa-miR-196a | 2,2E-02 |
| ENSG00000089820 | hsa-miR-200b* | 2,2E-02 |
| ENSG00000104213 | hsa-miR-151-3p | 2,2E-02 |
| ENSG00000185432 | hsa-miR-17 | 2,2E-02 |
| ENSG00000151623 | hsa-miR-151-5p | 2,2E-02 |
| ENSG00000134531 | hsa-miR-200b | 2,2E-02 |
| ENSG00000077942 | hsa-miR-375 | 2,2E-02 |
| ENSG00000168497 | hsa-miR-203 | 2,2E-02 |
| ENSG00000166670 | hsa-miR-100 | 2,2E-02 |
| ENSG00000055118 | hsa-miR-34a | 2,2E-02 |
| ENSG00000113916 | hsa-miR-30c | 2,2E-02 |
| ENSG00000172456 | hsa-miR-143 | 2,2E-02 |
| ENSG00000131459 | hsa-miR-30c | 2,2E-02 |
| ENSG00000105664 | hsa-miR-886-3p | 2,2E-02 |

| | | |
|---|---|---|
| ENSG00000206075 | hsa-miR-221 | 2,2E-02 |
| ENSG00000115163 | hsa-let-7g | 2,2E-02 |
| ENSG00000204086 | hsa-miR-149 | 2,2E-02 |
| ENSG00000133392 | hsa-miR-1238 | 2,2E-02 |
| ENSG00000136231 | hsa-miR-223 | 2,2E-02 |
| ENSG00000136231 | hsa-let-7e | 2,2E-02 |
| ENSG00000105519 | hsa-miR-197 | 2,2E-02 |
| ENSG00000167757 | hsa-miR-186 | 2,2E-02 |
| ENSG00000183578 | hsa-miR-497 | 2,3E-02 |
| ENSG00000118322 | hsa-miR-23b | 2,3E-02 |
| ENSG00000113368 | hsa-miR-23a | 2,3E-02 |
| ENSG00000144451 | hsa-miR-185 | 2,3E-02 |
| ENSG00000176083 | hsa-miR-125a-5p | 2,3E-02 |
| ENSG00000077274 | hsa-miR-574-5p | 2,3E-02 |
| ENSG00000144642 | hsa-miR-93 | 2,3E-02 |
| ENSG00000139055 | hsa-miR-424 | 2,3E-02 |
| ENSG00000166523 | hsa-let-7d | 2,3E-02 |
| ENSG00000112379 | hsa-miR-125b | 2,3E-02 |
| ENSG00000166033 | hsa-miR-320a | 2,3E-02 |
| ENSG00000168079 | hsa-miR-939 | 2,3E-02 |
| ENSG00000099984 | hsa-miR-16 | 2,3E-02 |
| ENSG00000026036 | hsa-miR-30e* | 2,3E-02 |
| ENSG00000170231 | hsa-miR-223 | 2,3E-02 |
| ENSG00000135046 | hsa-miR-196b | 2,3E-02 |
| ENSG00000158859 | hsa-miR-141 | 2,4E-02 |
| ENSG00000060982 | hsa-miR-96 | 2,4E-02 |
| ENSG00000198915 | hsa-miR-93 | 2,4E-02 |
| ENSG00000172164 | hsa-miR-21 | 2,4E-02 |
| ENSG00000017427 | hsa-miR-155 | 2,4E-02 |
| ENSG00000163421 | hsa-miR-494 | 2,4E-02 |
| ENSG00000114405 | hsa-miR-186 | 2,4E-02 |
| ENSG00000153234 | hsa-miR-93 | 2,4E-02 |
| ENSG00000158270 | hsa-miR-7 | 2,4E-02 |
| ENSG00000105976 | hsa-miR-451 | 2,4E-02 |
| ENSG00000073849 | hsa-miR-210 | 2,4E-02 |
| ENSG00000073712 | hsa-miR-29b | 2,4E-02 |
| ENSG00000000971 | hsa-miR-146a | 2,4E-02 |
| ENSG00000060982 | hsa-miR-150 | 2,4E-02 |
| ENSG00000172156 | hsa-miR-181b | 2,4E-02 |
| ENSG00000012504 | hsa-miR-20b | 2,4E-02 |
| ENSG00000005249 | hsa-miR-200b | 2,4E-02 |
| ENSG00000117394 | hsa-miR-185 | 2,4E-02 |
| ENSG00000168079 | hsa-miR-19b | 2,5E-02 |
| ENSG00000133392 | hsa-miR-17 | 2,5E-02 |
| ENSG00000138678 | hsa-miR-7 | 2,5E-02 |

| | | |
|---|---|---|
| ENSG00000134569 | hsa-miR-193a-3p | 2,5E-02 |
| ENSG00000175445 | hsa-miR-188-5p | 2,5E-02 |
| ENSG00000118515 | hsa-miR-200b* | 2,5E-02 |
| ENSG00000095739 | hsa-miR-1225-5p | 2,5E-02 |
| ENSG00000071794 | hsa-miR-141 | 2,5E-02 |
| ENSG00000162493 | hsa-miR-29c | 2,5E-02 |
| ENSG00000102265 | hsa-let-7g | 2,5E-02 |
| ENSG00000080031 | hsa-miR-663 | 2,5E-02 |
| ENSG00000137573 | hsa-miR-150 | 2,5E-02 |
| ENSG00000117394 | hsa-miR-140-5p | 2,5E-02 |
| ENSG00000198074 | hsa-miR-199b-5p | 2,5E-02 |
| ENSG00000134193 | hsa-miR-663 | 2,5E-02 |
| ENSG00000175920 | hsa-miR-663 | 2,5E-02 |
| ENSG00000082196 | hsa-miR-429 | 2,5E-02 |
| ENSG00000099953 | hsa-let-7d | 2,5E-02 |
| ENSG00000143387 | hsa-miR-107 | 2,5E-02 |
| ENSG00000121741 | hsa-miR-181b | 2,5E-02 |
| ENSG00000102678 | hsa-miR-130b | 2,5E-02 |
| ENSG00000130529 | hsa-miR-150 | 2,5E-02 |
| ENSG00000171766 | hsa-miR-22 | 2,5E-02 |
| ENSG00000129455 | hsa-miR-196b | 2,5E-02 |
| ENSG00000103707 | hsa-let-7i | 2,5E-02 |
| ENSG00000085831 | hsa-miR-497 | 2,6E-02 |
| ENSG00000119125 | hsa-miR-27a | 2,6E-02 |
| ENSG00000123096 | hsa-miR-93 | 2,6E-02 |
| ENSG00000026025 | hsa-let-7f | 2,6E-02 |
| ENSG00000062524 | hsa-miR-151-3p | 2,6E-02 |
| ENSG00000102010 | hsa-miR-15a | 2,6E-02 |
| ENSG00000163430 | hsa-miR-766 | 2,6E-02 |
| ENSG00000113303 | hsa-miR-451 | 2,6E-02 |
| ENSG00000103569 | hsa-miR-196a | 2,6E-02 |
| ENSG00000100234 | hsa-miR-766 | 2,6E-02 |
| ENSG00000164932 | hsa-miR-30e | 2,6E-02 |
| ENSG00000103534 | hsa-miR-143 | 2,6E-02 |
| ENSG00000113916 | hsa-miR-93 | 2,6E-02 |
| ENSG00000104213 | hsa-let-7f-1* | 2,6E-02 |
| ENSG00000114378 | hsa-miR-572 | 2,6E-02 |
| ENSG00000144642 | hsa-miR-96 | 2,6E-02 |
| ENSG00000166482 | hsa-miR-200a | 2,6E-02 |
| ENSG00000165617 | hsa-miR-93 | 2,6E-02 |
| ENSG00000103485 | hsa-miR-497 | 2,6E-02 |
| ENSG00000148926 | hsa-miR-497 | 2,6E-02 |
| ENSG00000111863 | hsa-miR-185 | 2,6E-02 |
| ENSG00000007171 | hsa-miR-497 | 2,6E-02 |
| ENSG00000170312 | hsa-miR-143 | 2,6E-02 |

| | | |
|---|---|---|
| ENSG00000116711 | hsa-miR-25 | 2,6E-02 |
| ENSG00000187134 | hsa-miR-365 | 2,6E-02 |
| ENSG00000036473 | hsa-miR-100 | 2,6E-02 |
| ENSG00000068078 | hsa-miR-29c | 2,6E-02 |
| ENSG00000050344 | hsa-miR-199a-5p | 2,6E-02 |
| ENSG00000181690 | hsa-miR-320a | 2,6E-02 |
| ENSG00000125775 | hsa-miR-497 | 2,6E-02 |
| ENSG00000141506 | hsa-miR-98 | 2,7E-02 |
| ENSG00000144045 | hsa-miR-301a | 2,7E-02 |
| ENSG00000105835 | hsa-miR-30a | 2,7E-02 |
| ENSG00000175471 | hsa-miR-532-5p | 2,7E-02 |
| ENSG00000077782 | hsa-miR-532-5p | 2,7E-02 |
| ENSG00000082196 | hsa-miR-1274a | 2,7E-02 |
| ENSG00000140450 | hsa-miR-25 | 2,7E-02 |
| ENSG00000166523 | hsa-miR-196b | 2,7E-02 |
| ENSG00000137573 | hsa-miR-1280 | 2,7E-02 |
| ENSG00000153234 | hsa-miR-19b | 2,7E-02 |
| ENSG00000142677 | hsa-miR-324-3p | 2,7E-02 |
| ENSG00000144642 | hsa-miR-7 | 2,7E-02 |
| ENSG00000160180 | hsa-miR-214 | 2,7E-02 |
| ENSG00000137634 | hsa-miR-146b-5p | 2,7E-02 |
| ENSG00000087303 | hsa-let-7f | 2,7E-02 |
| ENSG00000006118 | hsa-miR-203 | 2,7E-02 |
| ENSG00000129277 | hsa-miR-24 | 2,7E-02 |
| ENSG00000111907 | hsa-miR-25 | 2,7E-02 |
| ENSG00000017427 | hsa-miR-96 | 2,7E-02 |
| ENSG00000077063 | hsa-miR-140-5p | 2,7E-02 |
| ENSG00000128849 | hsa-miR-1228 | 2,7E-02 |
| ENSG00000170956 | hsa-miR-100 | 2,7E-02 |
| ENSG00000117594 | hsa-miR-30c | 2,7E-02 |
| ENSG00000102471 | hsa-miR-145 | 2,7E-02 |
| ENSG00000181690 | hsa-miR-362-5p | 2,7E-02 |
| ENSG00000178226 | hsa-miR-375 | 2,8E-02 |
| ENSG00000172367 | hsa-miR-130b | 2,8E-02 |
| ENSG00000188818 | hsa-miR-221 | 2,8E-02 |
| ENSG00000171812 | hsa-miR-320c | 2,8E-02 |
| ENSG00000122641 | hsa-miR-10b | 2,8E-02 |
| ENSG00000139926 | hsa-miR-150 | 2,8E-02 |
| ENSG00000068650 | hsa-miR-181b | 2,8E-02 |
| ENSG00000116785 | hsa-miR-590-5p | 2,8E-02 |
| ENSG00000088836 | hsa-miR-140-5p | 2,8E-02 |
| ENSG00000185269 | hsa-miR-210 | 2,8E-02 |
| ENSG00000177459 | hsa-miR-494 | 2,8E-02 |
| ENSG00000198523 | hsa-miR-425 | 2,8E-02 |
| ENSG00000171877 | hsa-miR-96 | 2,8E-02 |

| | | |
|---|---|---|
| ENSG00000169439 | hsa-miR-96 | 2,8E-02 |
| ENSG00000065534 | hsa-miR-625 | 2,8E-02 |
| ENSG00000179603 | hsa-miR-155 | 2,8E-02 |
| ENSG00000155760 | hsa-miR-142-3p | 2,8E-02 |
| ENSG00000153292 | hsa-miR-19b | 2,8E-02 |
| ENSG00000162493 | hsa-miR-151-5p | 2,8E-02 |
| ENSG00000101346 | hsa-miR-140-3p | 2,8E-02 |
| ENSG00000122257 | hsa-miR-625 | 2,8E-02 |
| ENSG00000206538 | hsa-miR-215 | 2,8E-02 |
| ENSG00000119714 | hsa-miR-221 | 2,8E-02 |
| ENSG00000173210 | hsa-miR-200a* | 2,8E-02 |
| ENSG00000132386 | hsa-miR-200a* | 2,8E-02 |
| ENSG00000135643 | hsa-miR-424 | 2,8E-02 |
| ENSG00000112096 | hsa-miR-98 | 2,9E-02 |
| ENSG00000106624 | hsa-miR-766 | 2,9E-02 |
| ENSG00000163486 | hsa-miR-223 | 2,9E-02 |
| ENSG00000180447 | hsa-miR-194 | 2,9E-02 |
| ENSG00000149089 | hsa-miR-338-3p | 2,9E-02 |
| ENSG00000007908 | hsa-miR-141 | 2,9E-02 |
| ENSG00000100453 | hsa-miR-375 | 2,9E-02 |
| ENSG00000101144 | hsa-miR-22 | 2,9E-02 |
| ENSG00000166923 | hsa-miR-425 | 2,9E-02 |
| ENSG00000125845 | hsa-miR-140-5p | 2,9E-02 |
| ENSG00000211935 | hsa-miR-200a | 2,9E-02 |
| ENSG00000123689 | hsa-miR-454 | 2,9E-02 |
| ENSG00000133110 | hsa-miR-183 | 2,9E-02 |
| ENSG00000152661 | hsa-miR-93 | 2,9E-02 |
| ENSG00000103044 | hsa-miR-26b | 2,9E-02 |
| ENSG00000130203 | hsa-let-7d | 2,9E-02 |
| ENSG00000161249 | hsa-miR-150* | 2,9E-02 |
| ENSG00000125869 | hsa-miR-320c | 2,9E-02 |
| ENSG00000007306 | hsa-miR-362-5p | 2,9E-02 |
| ENSG00000163347 | hsa-miR-625 | 2,9E-02 |
| ENSG00000123689 | hsa-miR-210 | 2,9E-02 |
| ENSG00000149582 | hsa-miR-625 | 3,0E-02 |
| ENSG00000064270 | hsa-miR-663 | 3,0E-02 |
| ENSG00000137752 | hsa-let-7b | 3,0E-02 |
| ENSG00000176194 | hsa-miR-29b | 3,0E-02 |
| ENSG00000178462 | hsa-miR-660 | 3,0E-02 |
| ENSG00000134569 | hsa-miR-320a | 3,0E-02 |
| ENSG00000147394 | hsa-miR-181b | 3,0E-02 |
| ENSG00000183578 | hsa-miR-15a | 3,0E-02 |
| ENSG00000120093 | hsa-miR-27a | 3,0E-02 |
| ENSG00000206075 | hsa-miR-1246 | 3,0E-02 |
| ENSG00000057019 | hsa-miR-378 | 3,0E-02 |

| | | |
|---|---|---|
| ENSG00000114251 | hsa-let-7b | 3,0E-02 |
| ENSG00000139874 | hsa-miR-125b | 3,0E-02 |
| ENSG00000179603 | hsa-miR-140-3p | 3,0E-02 |
| ENSG00000131746 | hsa-miR-30c | 3,0E-02 |
| ENSG00000103534 | hsa-miR-769-5p | 3,0E-02 |
| ENSG00000164292 | hsa-miR-142-3p | 3,0E-02 |
| ENSG00000120694 | hsa-miR-185 | 3,1E-02 |
| ENSG00000178878 | hsa-miR-625 | 3,1E-02 |
| ENSG00000113721 | hsa-miR-141 | 3,1E-02 |
| ENSG00000183844 | hsa-miR-155 | 3,1E-02 |
| ENSG00000181495 | hsa-miR-215 | 3,1E-02 |
| ENSG00000105519 | hsa-miR-663 | 3,1E-02 |
| ENSG00000148346 | hsa-miR-331-3p | 3,1E-02 |
| ENSG00000077782 | hsa-miR-1275 | 3,1E-02 |
| ENSG00000006016 | hsa-miR-766 | 3,1E-02 |
| ENSG00000135083 | hsa-miR-30a | 3,1E-02 |
| ENSG00000146477 | hsa-miR-210 | 3,1E-02 |
| ENSG00000103044 | hsa-miR-22 | 3,1E-02 |
| ENSG00000157193 | hsa-miR-100 | 3,1E-02 |
| ENSG00000181019 | hsa-miR-34a | 3,1E-02 |
| ENSG00000158859 | hsa-miR-940 | 3,1E-02 |
| ENSG00000138079 | hsa-miR-34a | 3,1E-02 |
| ENSG00000104783 | hsa-miR-497 | 3,1E-02 |
| ENSG00000130208 | hsa-miR-20b | 3,1E-02 |
| ENSG00000130513 | hsa-miR-29c | 3,1E-02 |
| ENSG00000155760 | hsa-miR-20b | 3,1E-02 |
| ENSG00000169903 | hsa-miR-126 | 3,1E-02 |
| ENSG00000169442 | hsa-miR-130b | 3,1E-02 |
| ENSG00000050344 | hsa-miR-223 | 3,1E-02 |
| ENSG00000129455 | hsa-miR-196a | 3,1E-02 |
| ENSG00000131203 | hsa-miR-148a | 3,1E-02 |
| ENSG00000169271 | hsa-miR-574-5p | 3,1E-02 |
| ENSG00000181804 | hsa-miR-203 | 3,1E-02 |
| ENSG00000151490 | hsa-miR-26b | 3,1E-02 |
| ENSG00000130513 | hsa-miR-22 | 3,2E-02 |
| ENSG00000181495 | hsa-miR-221 | 3,2E-02 |
| ENSG00000166825 | hsa-miR-10a | 3,2E-02 |
| ENSG00000130508 | hsa-miR-19b | 3,2E-02 |
| ENSG00000134215 | hsa-miR-181b | 3,2E-02 |
| ENSG00000119125 | hsa-let-7g | 3,2E-02 |
| ENSG00000130035 | hsa-miR-29b | 3,2E-02 |
| ENSG00000169429 | hsa-miR-20a | 3,2E-02 |
| ENSG00000110080 | hsa-miR-98 | 3,2E-02 |
| ENSG00000078114 | hsa-miR-19a | 3,2E-02 |
| ENSG00000113721 | hsa-miR-378 | 3,2E-02 |

| | | |
|---|---|---|
| ENSG00000206538 | hsa-miR-30e | 3,2E-02 |
| ENSG00000103044 | hsa-miR-10a | 3,2E-02 |
| ENSG00000006075 | hsa-let-7g | 3,2E-02 |
| ENSG00000117091 | hsa-miR-141 | 3,2E-02 |
| ENSG00000085662 | hsa-miR-196b | 3,2E-02 |
| ENSG00000158859 | hsa-miR-155 | 3,2E-02 |
| ENSG00000132329 | hsa-let-7d | 3,3E-02 |
| ENSG00000114113 | hsa-miR-625 | 3,3E-02 |
| ENSG00000164932 | hsa-let-7f | 3,3E-02 |
| ENSG00000065534 | hsa-miR-574-3p | 3,3E-02 |
| ENSG00000108786 | hsa-miR-93 | 3,3E-02 |
| ENSG00000110077 | hsa-miR-148a | 3,3E-02 |
| ENSG00000118407 | hsa-miR-338-3p | 3,3E-02 |
| ENSG00000124006 | hsa-miR-29b-1* | 3,3E-02 |
| ENSG00000049323 | hsa-miR-320c | 3,3E-02 |
| ENSG00000136997 | hsa-miR-210 | 3,3E-02 |
| ENSG00000104450 | hsa-miR-625 | 3,3E-02 |
| ENSG00000157404 | hsa-miR-142-3p | 3,3E-02 |
| ENSG00000101004 | hsa-miR-574-5p | 3,3E-02 |
| ENSG00000143882 | hsa-miR-148a | 3,3E-02 |
| ENSG00000173917 | hsa-miR-378 | 3,3E-02 |
| ENSG00000108244 | hsa-miR-301a | 3,3E-02 |
| ENSG00000151490 | hsa-miR-1275 | 3,3E-02 |
| ENSG00000110427 | hsa-miR-660 | 3,3E-02 |
| ENSG00000158813 | hsa-miR-638 | 3,4E-02 |
| ENSG00000152413 | hsa-miR-130b | 3,4E-02 |
| ENSG00000154822 | hsa-miR-532-5p | 3,4E-02 |
| ENSG00000173404 | hsa-miR-23b | 3,4E-02 |
| ENSG00000007952 | hsa-miR-23b | 3,4E-02 |
| ENSG00000185090 | hsa-let-7e | 3,4E-02 |
| ENSG00000079215 | hsa-miR-454 | 3,4E-02 |
| ENSG00000243244 | hsa-miR-107 | 3,4E-02 |
| ENSG00000118407 | hsa-miR-130b | 3,4E-02 |
| ENSG00000139926 | hsa-miR-301a | 3,4E-02 |
| ENSG00000064787 | hsa-miR-17 | 3,4E-02 |
| ENSG00000106025 | hsa-miR-18a | 3,4E-02 |
| ENSG00000104723 | hsa-miR-183 | 3,4E-02 |
| ENSG00000142875 | hsa-miR-34a | 3,4E-02 |
| ENSG00000141293 | hsa-miR-210 | 3,4E-02 |
| ENSG00000145824 | hsa-miR-625 | 3,4E-02 |
| ENSG00000065833 | hsa-miR-26a | 3,4E-02 |
| ENSG00000074527 | hsa-miR-98 | 3,4E-02 |
| ENSG00000136367 | hsa-miR-574-5p | 3,4E-02 |
| ENSG00000129474 | hsa-miR-15a | 3,4E-02 |
| ENSG00000117228 | hsa-miR-30c | 3,4E-02 |

| | | |
|---|---|---|
| ENSG00000176907 | hsa-miR-338-3p | 3,4E-02 |
| ENSG00000134871 | hsa-miR-210 | 3,4E-02 |
| ENSG00000197380 | hsa-miR-574-5p | 3,4E-02 |
| ENSG00000114378 | hsa-miR-17* | 3,5E-02 |
| ENSG00000120708 | hsa-miR-590-5p | 3,5E-02 |
| ENSG00000162779 | hsa-miR-625 | 3,5E-02 |
| ENSG00000134531 | hsa-miR-7 | 3,5E-02 |
| ENSG00000153936 | hsa-miR-15b | 3,5E-02 |
| ENSG00000133048 | hsa-let-7b | 3,5E-02 |
| ENSG00000144366 | hsa-miR-130b | 3,5E-02 |
| ENSG00000101333 | hsa-miR-29b-1* | 3,5E-02 |
| ENSG00000107796 | hsa-miR-590-5p | 3,5E-02 |
| ENSG00000187479 | hsa-miR-19b | 3,5E-02 |
| ENSG00000078596 | hsa-miR-96 | 3,5E-02 |
| ENSG00000105609 | hsa-miR-301a | 3,5E-02 |
| ENSG00000136231 | hsa-miR-196a | 3,5E-02 |
| ENSG00000157445 | hsa-miR-1246 | 3,5E-02 |
| ENSG00000165449 | hsa-miR-574-5p | 3,5E-02 |
| ENSG00000130508 | hsa-miR-194 | 3,5E-02 |
| ENSG00000198523 | hsa-miR-29b-1* | 3,5E-02 |
| ENSG00000113916 | hsa-miR-19b | 3,5E-02 |
| ENSG00000177283 | hsa-miR-663 | 3,6E-02 |
| ENSG00000182472 | hsa-miR-296-5p | 3,6E-02 |
| ENSG00000136235 | hsa-miR-183 | 3,6E-02 |
| ENSG00000115457 | hsa-miR-663 | 3,6E-02 |
| ENSG00000146021 | hsa-miR-19b | 3,6E-02 |
| ENSG00000182253 | hsa-miR-200c | 3,6E-02 |
| ENSG00000166831 | hsa-miR-939 | 3,6E-02 |
| ENSG00000148735 | hsa-miR-149 | 3,6E-02 |
| ENSG00000181458 | hsa-miR-19a | 3,6E-02 |
| ENSG00000152503 | hsa-miR-23a | 3,6E-02 |
| ENSG00000172986 | hsa-miR-18a | 3,6E-02 |
| ENSG00000197380 | hsa-miR-186 | 3,6E-02 |
| ENSG00000177575 | hsa-miR-141 | 3,6E-02 |
| ENSG00000145431 | hsa-miR-130b | 3,6E-02 |
| ENSG00000198523 | hsa-miR-215 | 3,6E-02 |
| ENSG00000058668 | hsa-miR-186 | 3,6E-02 |
| ENSG00000079385 | hsa-miR-146b-5p | 3,6E-02 |
| ENSG00000017427 | hsa-miR-148a | 3,6E-02 |
| ENSG00000154639 | hsa-miR-497 | 3,6E-02 |
| ENSG00000135245 | hsa-miR-17 | 3,6E-02 |
| ENSG00000163898 | hsa-miR-1246 | 3,6E-02 |
| ENSG00000064270 | hsa-miR-140-3p | 3,6E-02 |
| ENSG00000118515 | hsa-miR-15b | 3,6E-02 |
| ENSG00000130513 | hsa-miR-194 | 3,6E-02 |

| | | |
|---|---|---|
| ENSG00000041982 | hsa-miR-96 | 3,6E-02 |
| ENSG00000164120 | hsa-miR-17 | 3,7E-02 |
| ENSG00000117228 | hsa-miR-30e | 3,7E-02 |
| ENSG00000173404 | hsa-miR-203 | 3,7E-02 |
| ENSG00000148926 | hsa-miR-16 | 3,7E-02 |
| ENSG00000187860 | hsa-miR-22 | 3,7E-02 |
| ENSG00000138755 | hsa-miR-30a | 3,7E-02 |
| ENSG00000115009 | hsa-miR-143 | 3,7E-02 |
| ENSG00000113303 | hsa-miR-23a | 3,7E-02 |
| ENSG00000175899 | hsa-miR-590-5p | 3,7E-02 |
| ENSG00000149582 | hsa-miR-221 | 3,7E-02 |
| ENSG00000112494 | hsa-miR-210 | 3,7E-02 |
| ENSG00000197619 | hsa-miR-29b-1* | 3,7E-02 |
| ENSG00000131620 | hsa-miR-19b | 3,7E-02 |
| ENSG00000068650 | hsa-miR-494 | 3,7E-02 |
| ENSG00000138180 | hsa-miR-130a | 3,7E-02 |
| ENSG00000107562 | hsa-miR-28-5p | 3,7E-02 |
| ENSG00000102837 | hsa-miR-146b-5p | 3,7E-02 |
| ENSG00000123500 | hsa-miR-10a | 3,7E-02 |
| ENSG00000215182 | hsa-miR-20b | 3,7E-02 |
| ENSG00000244414 | hsa-miR-146a | 3,7E-02 |
| ENSG00000106236 | hsa-miR-96 | 3,7E-02 |
| ENSG00000104783 | hsa-miR-107 | 3,7E-02 |
| ENSG00000133878 | hsa-miR-15b | 3,7E-02 |
| ENSG00000173210 | hsa-miR-148a | 3,7E-02 |
| ENSG00000077942 | hsa-miR-155 | 3,7E-02 |
| ENSG00000116785 | hsa-miR-146a | 3,7E-02 |
| ENSG00000186198 | hsa-miR-29b-1* | 3,7E-02 |
| ENSG00000173404 | hsa-miR-26a | 3,7E-02 |
| ENSG00000181690 | hsa-miR-186 | 3,7E-02 |
| ENSG00000092621 | hsa-miR-23a | 3,8E-02 |
| ENSG00000145632 | hsa-miR-532-5p | 3,8E-02 |
| ENSG00000152661 | hsa-miR-19b | 3,8E-02 |
| ENSG00000070019 | hsa-let-7i | 3,8E-02 |
| ENSG00000162520 | hsa-miR-30e | 3,8E-02 |
| ENSG00000163083 | hsa-miR-19a | 3,8E-02 |
| ENSG00000116299 | hsa-let-7e | 3,8E-02 |
| ENSG00000102048 | hsa-miR-1225-3p | 3,8E-02 |
| ENSG00000148082 | hsa-miR-210 | 3,8E-02 |
| ENSG00000204389 | hsa-miR-19a | 3,8E-02 |
| ENSG00000157551 | hsa-miR-200b | 3,8E-02 |
| ENSG00000122257 | hsa-miR-92a | 3,8E-02 |
| ENSG00000177464 | hsa-miR-196b | 3,8E-02 |
| ENSG00000170873 | hsa-miR-320a | 3,8E-02 |
| ENSG00000183578 | hsa-miR-15b | 3,8E-02 |

| | | |
|---|---|---|
| ENSG00000120068 | hsa-miR-342-3p | 3,8E-02 |
| ENSG00000196155 | hsa-miR-324-3p | 3,8E-02 |
| ENSG00000140285 | hsa-miR-93 | 3,8E-02 |
| ENSG00000135678 | hsa-let-7b | 3,8E-02 |
| ENSG00000057149 | hsa-miR-185 | 3,8E-02 |
| ENSG00000134215 | hsa-let-7d | 3,8E-02 |
| ENSG00000135218 | hsa-miR-93 | 3,8E-02 |
| ENSG00000100078 | hsa-miR-320c | 3,8E-02 |
| ENSG00000164114 | hsa-miR-15b | 3,8E-02 |
| ENSG00000117016 | hsa-miR-376c | 3,8E-02 |
| ENSG00000129455 | hsa-miR-20b | 3,8E-02 |
| ENSG00000117394 | hsa-miR-186 | 3,9E-02 |
| ENSG00000089177 | hsa-miR-30d | 3,9E-02 |
| ENSG00000197249 | hsa-miR-663 | 3,9E-02 |
| ENSG00000198019 | hsa-miR-375 | 3,9E-02 |
| ENSG00000104290 | hsa-miR-365 | 3,9E-02 |
| ENSG00000081237 | hsa-miR-203 | 3,9E-02 |
| ENSG00000136826 | hsa-miR-429 | 3,9E-02 |
| ENSG00000179344 | hsa-miR-769-5p | 3,9E-02 |
| ENSG00000113916 | hsa-miR-28-5p | 3,9E-02 |
| ENSG00000132465 | hsa-miR-574-5p | 3,9E-02 |
| ENSG00000109472 | hsa-miR-30d | 3,9E-02 |
| ENSG00000135842 | hsa-miR-1275 | 3,9E-02 |
| ENSG00000100234 | hsa-miR-15a | 3,9E-02 |
| ENSG00000206066 | hsa-miR-210 | 3,9E-02 |
| ENSG00000143184 | hsa-miR-378 | 3,9E-02 |
| ENSG00000164949 | hsa-miR-200c | 3,9E-02 |
| ENSG00000239839 | hsa-miR-143 | 3,9E-02 |
| ENSG00000168903 | hsa-miR-23b | 3,9E-02 |
| ENSG00000154721 | hsa-miR-19a | 3,9E-02 |
| ENSG00000100078 | hsa-miR-19b | 3,9E-02 |
| ENSG00000135447 | hsa-miR-17 | 3,9E-02 |
| ENSG00000184557 | hsa-miR-30e | 3,9E-02 |
| ENSG00000091138 | hsa-let-7c | 3,9E-02 |
| ENSG00000177464 | hsa-miR-148a | 3,9E-02 |
| ENSG00000136997 | hsa-miR-146a | 3,9E-02 |
| ENSG00000184185 | hsa-miR-296-5p | 3,9E-02 |
| ENSG00000006074 | hsa-miR-194* | 4,0E-02 |
| ENSG00000159182 | hsa-miR-221 | 4,0E-02 |
| ENSG00000101938 | hsa-miR-1246 | 4,0E-02 |
| ENSG00000117289 | hsa-miR-374a | 4,0E-02 |
| ENSG00000142871 | hsa-miR-155 | 4,0E-02 |
| ENSG00000054983 | hsa-miR-186 | 4,0E-02 |
| ENSG00000121297 | hsa-miR-301a | 4,0E-02 |
| ENSG00000141506 | hsa-miR-194* | 4,0E-02 |

| | | |
|---|---|---|
| ENSG00000154734 | hsa-miR-20a | 4,0E-02 |
| ENSG00000136231 | hsa-miR-320a | 4,0E-02 |
| ENSG00000203805 | hsa-miR-142-3p | 4,0E-02 |
| ENSG00000169876 | hsa-miR-769-5p | 4,0E-02 |
| ENSG00000055950 | hsa-miR-378 | 4,0E-02 |
| ENSG00000172724 | hsa-miR-1274b | 4,0E-02 |
| ENSG00000170425 | hsa-miR-30d | 4,0E-02 |
| ENSG00000080823 | hsa-miR-574-3p | 4,0E-02 |
| ENSG00000183734 | hsa-miR-338-3p | 4,0E-02 |
| ENSG00000133169 | hsa-miR-200a* | 4,0E-02 |
| ENSG00000125845 | hsa-miR-24 | 4,0E-02 |
| ENSG00000167748 | hsa-let-7f | 4,0E-02 |
| ENSG00000177459 | hsa-miR-425 | 4,0E-02 |
| ENSG00000145569 | hsa-miR-140-3p | 4,0E-02 |
| ENSG00000151490 | hsa-miR-940 | 4,0E-02 |
| ENSG00000065717 | hsa-miR-29b-1* | 4,0E-02 |
| ENSG00000158467 | hsa-miR-34a | 4,0E-02 |
| ENSG00000043462 | hsa-miR-20b | 4,1E-02 |
| ENSG00000198467 | hsa-let-7f | 4,1E-02 |
| ENSG00000104267 | hsa-miR-374a | 4,1E-02 |
| ENSG00000135750 | hsa-miR-148b | 4,1E-02 |
| ENSG00000169604 | hsa-miR-1275 | 4,1E-02 |
| ENSG00000166165 | hsa-miR-15a | 4,1E-02 |
| ENSG00000083807 | hsa-miR-324-3p | 4,1E-02 |
| ENSG00000078098 | hsa-miR-30b | 4,1E-02 |
| ENSG00000154556 | hsa-miR-146a | 4,1E-02 |
| ENSG00000129993 | hsa-miR-663 | 4,1E-02 |
| ENSG00000174469 | hsa-miR-185 | 4,1E-02 |
| ENSG00000105063 | hsa-miR-181a | 4,1E-02 |
| ENSG00000120093 | hsa-miR-1268 | 4,1E-02 |
| ENSG00000117594 | hsa-miR-192 | 4,1E-02 |
| ENSG00000151623 | hsa-miR-532-5p | 4,1E-02 |
| ENSG00000185847 | hsa-miR-28-5p | 4,1E-02 |
| ENSG00000139055 | hsa-miR-210 | 4,1E-02 |
| ENSG00000198467 | hsa-miR-155 | 4,1E-02 |
| ENSG00000110799 | hsa-miR-107 | 4,1E-02 |
| ENSG00000157315 | hsa-miR-214 | 4,1E-02 |
| ENSG00000158528 | hsa-miR-140-5p | 4,1E-02 |
| ENSG00000107562 | hsa-miR-155 | 4,1E-02 |
| ENSG00000198915 | hsa-miR-1281 | 4,1E-02 |
| ENSG00000123095 | hsa-miR-93 | 4,1E-02 |
| ENSG00000180376 | hsa-miR-10a | 4,1E-02 |
| ENSG00000133169 | hsa-miR-194 | 4,2E-02 |
| ENSG00000079215 | hsa-miR-130b | 4,2E-02 |
| ENSG00000167772 | hsa-miR-331-3p | 4,2E-02 |

| | | |
|---|---|---|
| ENSG00000197381 | hsa-miR-1207-5p | 4,2E-02 |
| ENSG00000205809 | hsa-miR-125b | 4,2E-02 |
| ENSG00000137673 | hsa-miR-130b | 4,2E-02 |
| ENSG00000134215 | hsa-miR-34a | 4,2E-02 |
| ENSG00000150594 | hsa-miR-25 | 4,2E-02 |
| ENSG00000168785 | hsa-miR-15a | 4,2E-02 |
| ENSG00000168542 | hsa-miR-96 | 4,2E-02 |
| ENSG00000206075 | hsa-miR-1202 | 4,2E-02 |
| ENSG00000172201 | hsa-miR-19b | 4,2E-02 |
| ENSG00000108576 | hsa-miR-16 | 4,2E-02 |
| ENSG00000168497 | hsa-miR-454 | 4,2E-02 |
| ENSG00000007908 | hsa-miR-221 | 4,2E-02 |
| ENSG00000173262 | hsa-miR-30b | 4,2E-02 |
| ENSG00000157470 | hsa-miR-30c | 4,2E-02 |
| ENSG00000103888 | hsa-miR-29c | 4,2E-02 |
| ENSG00000159261 | hsa-miR-30e | 4,2E-02 |
| ENSG00000150594 | hsa-miR-200a | 4,2E-02 |
| ENSG00000169509 | hsa-miR-625 | 4,2E-02 |
| ENSG00000117289 | hsa-miR-320b | 4,2E-02 |
| ENSG00000130508 | hsa-miR-454 | 4,3E-02 |
| ENSG00000141293 | hsa-miR-196b | 4,3E-02 |
| ENSG00000160712 | hsa-let-7b | 4,3E-02 |
| ENSG00000111087 | hsa-miR-200b | 4,3E-02 |
| ENSG00000132437 | hsa-miR-140-3p | 4,3E-02 |
| ENSG00000082196 | hsa-miR-494 | 4,3E-02 |
| ENSG00000077782 | hsa-miR-188-5p | 4,3E-02 |
| ENSG00000079215 | hsa-miR-194 | 4,3E-02 |
| ENSG00000132970 | hsa-miR-200c | 4,3E-02 |
| ENSG00000106541 | hsa-miR-125b | 4,3E-02 |
| ENSG00000026025 | hsa-miR-320c | 4,3E-02 |
| ENSG00000171812 | hsa-miR-196b | 4,3E-02 |
| ENSG00000170801 | hsa-miR-301a | 4,3E-02 |
| ENSG00000122786 | hsa-miR-320c | 4,3E-02 |
| ENSG00000171004 | hsa-miR-494 | 4,3E-02 |
| ENSG00000134569 | hsa-miR-455-3p | 4,3E-02 |
| ENSG00000115363 | hsa-miR-29c | 4,3E-02 |
| ENSG00000120594 | hsa-miR-10a | 4,3E-02 |
| ENSG00000211949 | hsa-miR-532-5p | 4,3E-02 |
| ENSG00000175445 | hsa-miR-215 | 4,3E-02 |
| ENSG00000157470 | hsa-miR-99b | 4,3E-02 |
| ENSG00000168243 | hsa-miR-183 | 4,3E-02 |
| ENSG00000148082 | hsa-let-7f | 4,3E-02 |
| ENSG00000102265 | hsa-miR-141 | 4,3E-02 |
| ENSG00000197991 | hsa-miR-98 | 4,3E-02 |
| ENSG00000147408 | hsa-miR-19a | 4,3E-02 |

| | | |
|---|---|---|
| ENSG00000015413 | hsa-miR-214 | 4,3E-02 |
| ENSG00000166147 | hsa-miR-20a | 4,4E-02 |
| ENSG00000156113 | hsa-miR-221 | 4,4E-02 |
| ENSG00000060140 | hsa-miR-376c | 4,4E-02 |
| ENSG00000119042 | hsa-miR-22 | 4,4E-02 |
| ENSG00000154175 | hsa-miR-425 | 4,4E-02 |
| ENSG00000163421 | hsa-miR-200c | 4,4E-02 |
| ENSG00000132437 | hsa-miR-185 | 4,4E-02 |
| ENSG00000162779 | hsa-miR-101 | 4,4E-02 |
| ENSG00000103569 | hsa-miR-192* | 4,4E-02 |
| ENSG00000123610 | hsa-miR-19b | 4,4E-02 |
| ENSG00000169902 | hsa-miR-194 | 4,4E-02 |
| ENSG00000135245 | hsa-miR-21* | 4,4E-02 |
| ENSG00000100290 | hsa-miR-424 | 4,4E-02 |
| ENSG00000073756 | hsa-miR-17 | 4,4E-02 |
| ENSG00000188343 | hsa-miR-149 | 4,4E-02 |
| ENSG00000104783 | hsa-miR-155 | 4,4E-02 |
| ENSG00000204262 | hsa-miR-130b | 4,4E-02 |
| ENSG00000167244 | hsa-miR-200a | 4,4E-02 |
| ENSG00000115956 | hsa-miR-141 | 4,4E-02 |
| ENSG00000147027 | hsa-miR-200c | 4,4E-02 |
| ENSG00000172215 | hsa-miR-199a-5p | 4,4E-02 |
| ENSG00000116741 | hsa-miR-221 | 4,4E-02 |
| ENSG00000146592 | hsa-miR-188-5p | 4,4E-02 |
| ENSG00000134775 | hsa-miR-215 | 4,4E-02 |
| ENSG00000177283 | hsa-miR-1275 | 4,4E-02 |
| ENSG00000087303 | hsa-miR-29b | 4,4E-02 |
| ENSG00000197381 | hsa-miR-1260 | 4,4E-02 |
| ENSG00000095587 | hsa-miR-342-3p | 4,5E-02 |
| ENSG00000153563 | hsa-miR-103 | 4,5E-02 |
| ENSG00000055950 | hsa-miR-142-3p | 4,5E-02 |
| ENSG00000177459 | hsa-miR-140-3p | 4,5E-02 |
| ENSG00000118407 | hsa-miR-378 | 4,5E-02 |
| ENSG00000162174 | hsa-miR-26b | 4,5E-02 |
| ENSG00000171208 | hsa-miR-23a | 4,5E-02 |
| ENSG00000078098 | hsa-miR-200c | 4,5E-02 |
| ENSG00000223609 | hsa-miR-98 | 4,5E-02 |
| ENSG00000165449 | hsa-let-7e | 4,5E-02 |
| ENSG00000187134 | hsa-miR-223 | 4,5E-02 |
| ENSG00000166482 | hsa-miR-25 | 4,5E-02 |
| ENSG00000142677 | hsa-miR-29c | 4,5E-02 |
| ENSG00000215182 | hsa-miR-29a | 4,5E-02 |
| ENSG00000110848 | hsa-miR-181a | 4,5E-02 |
| ENSG00000130830 | hsa-miR-200a* | 4,5E-02 |
| ENSG00000000971 | hsa-miR-200b | 4,5E-02 |

| | | |
|---|---|---|
| ENSG00000026036 | hsa-miR-188-5p | 4,5E-02 |
| ENSG00000188153 | hsa-let-7i | 4,5E-02 |
| ENSG00000167244 | hsa-miR-1234 | 4,5E-02 |
| ENSG00000094804 | hsa-miR-199b-5p | 4,5E-02 |
| ENSG00000129521 | hsa-miR-20a | 4,6E-02 |
| ENSG00000101955 | hsa-miR-494 | 4,6E-02 |
| ENSG00000152503 | hsa-miR-663 | 4,6E-02 |
| ENSG00000166670 | hsa-miR-181b | 4,6E-02 |
| ENSG00000206538 | hsa-miR-186 | 4,6E-02 |
| ENSG00000131389 | hsa-miR-497 | 4,6E-02 |
| ENSG00000129514 | hsa-miR-20a | 4,6E-02 |
| ENSG00000121297 | hsa-miR-200b | 4,6E-02 |
| ENSG00000147408 | hsa-miR-30d | 4,6E-02 |
| ENSG00000130208 | hsa-miR-93 | 4,6E-02 |
| ENSG00000151490 | hsa-miR-625 | 4,6E-02 |
| ENSG00000101187 | hsa-miR-30a | 4,6E-02 |
| ENSG00000133878 | hsa-miR-1275 | 4,6E-02 |
| ENSG00000159231 | hsa-miR-376c | 4,6E-02 |
| ENSG00000136982 | hsa-miR-454 | 4,6E-02 |
| ENSG00000149582 | hsa-miR-19b | 4,6E-02 |
| ENSG00000163347 | hsa-miR-199a-5p | 4,6E-02 |
| ENSG00000167748 | hsa-miR-638 | 4,6E-02 |
| ENSG00000154556 | hsa-miR-1246 | 4,6E-02 |
| ENSG00000112379 | hsa-miR-215 | 4,7E-02 |
| ENSG00000187908 | hsa-miR-145 | 4,7E-02 |
| ENSG00000106624 | hsa-miR-141 | 4,7E-02 |
| ENSG00000100450 | hsa-miR-196a | 4,7E-02 |
| ENSG00000198915 | hsa-miR-19b | 4,7E-02 |
| ENSG00000122861 | hsa-miR-183 | 4,7E-02 |
| ENSG00000110013 | hsa-miR-769-5p | 4,7E-02 |
| ENSG00000152518 | hsa-miR-155 | 4,7E-02 |
| ENSG00000043462 | hsa-miR-365 | 4,7E-02 |
| ENSG00000082196 | hsa-miR-7 | 4,7E-02 |
| ENSG00000130513 | hsa-miR-193b | 4,7E-02 |
| ENSG00000168078 | hsa-miR-192* | 4,7E-02 |
| ENSG00000223609 | hsa-miR-362-5p | 4,7E-02 |
| ENSG00000143333 | hsa-miR-19a | 4,7E-02 |
| ENSG00000087916 | hsa-miR-1228 | 4,7E-02 |
| ENSG00000125798 | hsa-miR-1246 | 4,7E-02 |
| ENSG00000170873 | hsa-miR-221 | 4,7E-02 |
| ENSG00000167434 | hsa-miR-939 | 4,7E-02 |
| ENSG00000101955 | hsa-miR-454 | 4,7E-02 |
| ENSG00000185432 | hsa-miR-194 | 4,7E-02 |
| ENSG00000181804 | hsa-miR-374a | 4,7E-02 |
| ENSG00000110013 | hsa-miR-125b | 4,7E-02 |

| | | |
|---|---|---|
| ENSG00000139874 | hsa-miR-27a | 4,7E-02 |
| ENSG00000178462 | hsa-miR-375 | 4,7E-02 |
| ENSG00000074410 | hsa-miR-342-3p | 4,7E-02 |
| ENSG00000101333 | hsa-miR-23b | 4,7E-02 |
| ENSG00000138755 | hsa-miR-149 | 4,8E-02 |
| ENSG00000102445 | hsa-miR-365 | 4,8E-02 |
| ENSG00000104267 | hsa-miR-30d | 4,8E-02 |
| ENSG00000153234 | hsa-miR-101 | 4,8E-02 |
| ENSG00000107968 | hsa-miR-20b | 4,8E-02 |
| ENSG00000132554 | hsa-miR-214 | 4,8E-02 |
| ENSG00000149968 | hsa-miR-183 | 4,8E-02 |
| ENSG00000122176 | hsa-miR-1207-5p | 4,8E-02 |
| ENSG00000171004 | hsa-miR-200a | 4,8E-02 |
| ENSG00000117289 | hsa-miR-939 | 4,8E-02 |
| ENSG00000082196 | hsa-miR-96 | 4,8E-02 |
| ENSG00000107968 | hsa-miR-494 | 4,8E-02 |
| ENSG00000165092 | hsa-miR-572 | 4,8E-02 |
| ENSG00000125869 | hsa-miR-494 | 4,8E-02 |
| ENSG00000171298 | hsa-miR-185 | 4,8E-02 |
| ENSG00000171208 | hsa-miR-532-5p | 4,8E-02 |
| ENSG00000111432 | hsa-miR-15b | 4,8E-02 |
| ENSG00000184292 | hsa-miR-195 | 4,8E-02 |
| ENSG00000115163 | hsa-let-7e | 4,8E-02 |
| ENSG00000163430 | hsa-miR-1249 | 4,8E-02 |
| ENSG00000117525 | hsa-miR-20a | 4,8E-02 |
| ENSG00000106025 | hsa-miR-140-5p | 4,8E-02 |
| ENSG00000100294 | hsa-let-7e | 4,8E-02 |
| ENSG00000139629 | hsa-miR-22 | 4,8E-02 |
| ENSG00000164692 | hsa-miR-186 | 4,8E-02 |
| ENSG00000151623 | hsa-miR-92a | 4,8E-02 |
| ENSG00000018625 | hsa-miR-93 | 4,8E-02 |
| ENSG00000073756 | hsa-miR-96 | 4,8E-02 |
| ENSG00000147883 | hsa-miR-572 | 4,9E-02 |
| ENSG00000137331 | hsa-miR-215 | 4,9E-02 |
| ENSG00000146054 | hsa-miR-638 | 4,9E-02 |
| ENSG00000136943 | hsa-let-7c | 4,9E-02 |
| ENSG00000103056 | hsa-miR-939 | 4,9E-02 |
| ENSG00000129654 | hsa-miR-10b | 4,9E-02 |
| ENSG00000172023 | hsa-miR-145 | 4,9E-02 |
| ENSG00000165244 | hsa-miR-16 | 4,9E-02 |
| ENSG00000107796 | hsa-miR-107 | 4,9E-02 |
| ENSG00000164114 | hsa-miR-494 | 4,9E-02 |
| ENSG00000164308 | hsa-miR-574-3p | 4,9E-02 |
| ENSG00000164761 | hsa-miR-21 | 4,9E-02 |
| ENSG00000140285 | hsa-miR-378 | 4,9E-02 |

| | | |
|---|---|---|
| ENSG00000086696 | hsa-miR-145 | 4,9E-02 |
| ENSG00000110079 | hsa-miR-93 | 4,9E-02 |
| ENSG00000176907 | hsa-miR-497 | 4,9E-02 |
| ENSG00000112379 | hsa-miR-125a-5p | 4,9E-02 |
| ENSG00000117016 | hsa-miR-141 | 4,9E-02 |
| ENSG00000156966 | hsa-miR-939 | 5,0E-02 |
| ENSG00000163735 | hsa-miR-199a-5p | 5,0E-02 |
| ENSG00000211611 | hsa-miR-200b* | 5,0E-02 |
| ENSG00000078401 | hsa-miR-625 | 5,0E-02 |
| ENSG00000064787 | hsa-miR-1207-5p | 5,0E-02 |
| ENSG00000113303 | hsa-miR-23b | 5,0E-02 |
| ENSG00000050405 | hsa-miR-93 | 5,0E-02 |
| ENSG00000165188 | hsa-miR-99b | 5,0E-02 |
| ENSG00000198959 | hsa-miR-29c | 5,0E-02 |
| ENSG00000188175 | hsa-miR-98 | 5,0E-02 |
| ENSG00000054983 | hsa-miR-1274b | 5,0E-02 |
| ENSG00000135773 | hsa-miR-126 | 5,0E-02 |
| ENSG00000104723 | hsa-miR-143 | 5,0E-02 |
| ENSG00000086289 | hsa-miR-28-5p | 5,0E-02 |
| ENSG00000087495 | hsa-miR-200c | 5,0E-02 |
| ENSG00000130830 | hsa-miR-203 | 5,0E-02 |
| ENSG00000138755 | hsa-miR-429 | 5,0E-02 |
| ENSG00000175538 | hsa-miR-196b | 5,0E-02 |
| ENSG00000110080 | hsa-let-7g | 5,0E-02 |
| ENSG00000122861 | hsa-let-7a | 5,0E-02 |
| ENSG00000145423 | hsa-miR-574-5p | 5,0E-02 |
| ENSG00000154639 | hsa-miR-25 | 5,0E-02 |
