# Supplementary material for: Molecular Classification of Colorectal Cancer by microRNA Profiling: Correlation with the Consensus Molecular Subtypes (CMS) and Validation of miR-30b Targets
Source: Cancers (Basel). 2022 Oct 22;14(21):5175. doi: 10.3390/cancers14215175 (PMC9656292; doi:10.3390/cancers14215175)
Supplement: Supplementary file 1 [file cancers-14-05175-s001.zip › cancers-1966190-supplementary/Supplementary Table S5.pdf]

Supplementary Table S5. Topological parameters of regulatory networks.

	Low Stroma	High Stroma	Mucinous
**Connected components**	1	1	1
**N of nodes**	250	234	89
**N of edges**	316	290	93
**Diameter**	18	18	17
**Radius**	10	9	9
**Characteristic path length**	6.294	6.747	7.677
**Avg num of neighbors**	2.528	2.479	2.090
**Centralization**	0.038	0.041	0.057
**Avg closeness**	0.164	0.154	0.136
**Avg radiality**	0.706	0.681	0.607

Topological parameters of the resulting networks for each miR subtype, considering only the largest connected component. In the network, miR and mRNAs are represented as nodes and the interaction between them as edges. Diameter and radius refer to the node eccentricity, while the characteristic path length is the average of the shortest path between every pair of nodes in the network. Average closeness centrality and average radially are both parameters that reflect the interconnection of the nodes within the network.
