# Supplementary material for: Molecular Classification of Colorectal Cancer by microRNA Profiling: Correlation with the Consensus Molecular Subtypes (CMS) and Validation of miR-30b Targets
Source: Cancers (Basel). 2022 Oct 22;14(21):5175. doi: 10.3390/cancers14215175 (PMC9656292; doi:10.3390/cancers14215175)
Supplement: Supplementary file 1 [file cancers-14-05175-s001.zip › cancers-1966190-supplementary/Supplementary Table S6.pdf]

Supplementary Table S6. Final interactions

Gene	microRNA	WSP score	LRS score	Microt score	PITA score	Microcosm score
FAP	hsa-miR-30b	0,237	0,742	-	-3,66	16,487
SPARC	hsa-miR-29b	0,101	0,666	-	-	-
SLC6A6	hsa-miR-30b	0,093	0,606	-	0,15	-
VIM	hsa-miR-30c	0,091	0,467	-	-	-
THBS2	hsa-miR-96	0,081	0,523	-	-	-
TRPM6	hsa-let-7c	0,080	0,618	0,796	-	-
VIM	hsa-miR-320c	0,073	0,402	0,858	-8,08	-
IL8	hsa-miR-20a	0,070	0,660	-	-1,34	-
G0S2	hsa-miR-454	0,059	0,546	-	-	15,812
IL1B	hsa-miR-590-5p	0,048	0,405	0,792	-	15,701
PLCB4	hsa-miR-23b	0,047	0,435	-	-7,72	-
SLC6A6	hsa-miR-200c	0,041	0,463	-	-	-
DCN	hsa-miR-7	0,036	0,296	-	-	17,212
DCN	hsa-miR-146a	0,035	0,447	-	-	15,653
OLFM4	hsa-miR-22	0,035	0,420	-	-	-
INHBA	hsa-miR-150	0,035	0,451	-	-	16,823
CXCL9	hsa-miR-429	0,034	0,252	0,676	-	-
INHBA	hsa-miR-93	0,032	0,401	-	-4,31	-
FAP	hsa-miR-200c	0,031	0,275	-	-	15,499
MMP7	hsa-miR-130b	0,030	0,293	-	-	15,919
SFRP4	hsa-miR-96	0,030	0,327	-	-	15,405
COMP	hsa-miR-886-3p	0,030	0,303	-	-	15,089
CXCL12	hsa-miR-28-5p	0,029	0,418	0,559	-	15,902
REG4	hsa-miR-378	0,028	0,262	-	-	16,417
REG4	hsa-miR-378	0,028	0,262	-	-	16,417
TIMP3	hsa-miR-940	0,028	0,210	0,798	-	-

ITGB2	hsa-miR-96	0,028	0,404	-	-	16,432
CXCL9	hsa-miR-320c	0,028	0,330	0,579	-	-
G0S2	hsa-miR-210	0,027	0,283	-	-	16,221
CXCL12	hsa-miR-103	0,026	0,268	-	-	15,931
OLFM4	hsa-miR-146b-5p	0,026	0,371	0,536	-	-
ASCL2	hsa-miR-338-3p	0,025	0,238	0,582	-	-
FAP	hsa-miR-429	0,025	0,239	-	-	15,805
OSM	hsa-let-7g	0,025	0,371	-	-	15,923
SLC6A6	hsa-miR-497	0,025	0,234	-	-	-
HSD17B2	hsa-miR-145	0,025	0,240	-	-	17,649
THBS2	hsa-miR-29b-1*	0,025	0,240	-	-	18,094
DUSP26	hsa-miR-15b	0,025	0,197	-	-	-
INHBA	hsa-miR-574-5p	0,025	0,240	0,899	-	16,002
DDC	hsa-miR-22	0,024	0,212	-	-	16,146
REG4	hsa-miR-181b	0,024	0,360	-	-	16,365
SPARC	hsa-miR-625	0,024	0,165	-	-	-
ST6GAL1	hsa-miR-185	0,024	0,329	-	-	-
PDGFRL	hsa-miR-151-3p	0,024	0,223	-	-	16,651
TIMP3	hsa-miR-766	0,024	0,211	-	-	-
ITGB2	hsa-miR-29b	0,024	0,240	-	-	16,873
INHBA	hsa-miR-10b	0,024	0,240	-	-	16,214
DDC	hsa-miR-140-3p	0,024	0,200	0,842	-	16,282
VIM	hsa-let-7f	0,024	0,289	-	-	-
OLFM4	hsa-miR-151-5p	0,024	0,194	-	-	15,990
CXCL9	hsa-miR-455-3p	0,024	0,172	-	-	-
AQP9	hsa-miR-192*	0,024	0,241	-	-	15,819
SLC3A1	hsa-miR-34a	0,023	0,282	-	-	16,073
WNT11	hsa-miR-21*	0,023	0,241	-	-	15,794
CAPN12	hsa-miR-296-5p	0,023	0,182	0,477	-	-
CAPN12	hsa-miR-338-3p	0,023	0,213	-	-	-

SPARC	hsa-miR-186	0,023	0,199	-	-	-
CXCL9	hsa-miR-149	0,023	0,199	-	-	-
SPARC	hsa-miR-1275	0,023	0,050	0,734	-	-
CXCL12	hsa-miR-155	0,023	0,000	-	-	-
VIM	hsa-miR-17*	0,023	0,000	-	-	17,546
ST6GAL 1	hsa-miR-210	0,000	0,000	-	-	-
VIM	hsa-miR-93	0,000	0,000	-	-	-
HSD17B 2	hsa-miR-223	0,000	0,000	-	-	-
TIMP3	hsa-miR-15a	0,000	0,000	-	-	-
PLCB4	hsa-miR-155	0,000	0,000	-	-	-
PLCB4	hsa-miR-939	0,000	0,000	-	-	-
AQP9	hsa-miR-196a	0,000	0,000	-	-	-
DDC	hsa-miR-185	0,000	0,000	-	-	-
DDC	hsa-miR-34a	0,000	0,000	-	-	-
DUSP26	hsa-miR-1275	0,000	0,000	-	-	-
REG4	hsa-miR-663	0,000	0,000	-	-	-
CXCL9	hsa-miR-30a	0,000	0,000	-	-	-
CXCL9	hsa-miR-30e	0,000	0,000	-	-	-
SLIT2	hsa-miR-200c	0,000	0,000	-	-	-
SLC26A2	hsa-let-7c	0,000	0,000	-	-	-
CCL19	hsa-miR-1274b	0,000	0,000	-	-	-
GAS1	hsa-miR-194	0,000	0,000	-	-	-
GAS1	hsa-miR-19a	0,000	0,000	-	-	-
GAS1	hsa-miR-93	0,000	0,000	-	-	-
CAPN12	hsa-miR-7	0,000	0,000	-	-	-
DMBT1	hsa-miR-145	0,000	0,000	-	-	-
PLCB4	hsa-miR-29b-1*	0,000	0,000	-	-	18,471
SLIT3	hsa-miR-200b	0,000	0,000	-	-	16,158
DDC	hsa-miR-24-1*	0,000	0,000	-	-	16,032
VIM	hsa-miR-96	0,000	0,000	-	-	15,994

FABP6	hsa-miR-223	0,000	0,000	-	-	15,691
PDGFRL	hsa-let-7f-1*	0,000	0,000	-	-	15,542
